# Synthesis of New Cationic Dicephalic Surfactants and Their Nonequivalent Adsorption at the Air/Solution Interface

**DOI:** 10.1021/acs.langmuir.4c04803

**Published:** 2025-03-21

**Authors:** Łukasz Lamch, Izabella Leszczyńska, Daria Długowska, Weronika Szczęsna - Górniak, Piotr Batys, Ewelina Jarek, Kazimiera A. Wilk, Piotr Warszyński

**Affiliations:** a Department of Organic and Pharmaceutical Technology, Faculty of Chemistry, Wrocław University of Science and Technology, Wybrzeże Wyspiańskiego 27, Wrocław 50-370, Poland; b Jerzy Haber Institute of Catalysis and Surface Chemistry, Polish Academy of Sciences, Niezapominajek 8, Kraków 30-239, Poland

## Abstract

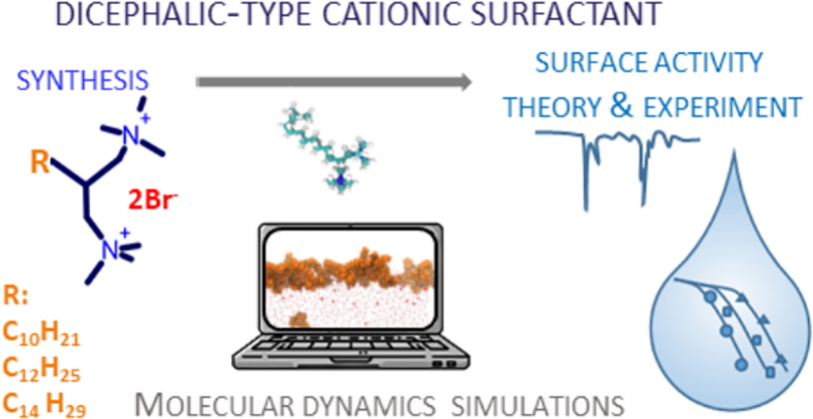

The interfacial behavior of aqueous solutions of newly synthesized 2-alkyl-*N*,*N*,*N*,*N*′,*N*′,*N*′-hexamethylpropan-1,3-ammonium dibromides with decyl, dodecyl, and tetradecyl alkyl chains was investigated both experimentally and theoretically. The results of the surface tension measurements were described using the modified surface quasi-two-dimensional electrolyte (mSTDE) model of ionic surfactant adsorption, which was supported by molecular dynamics simulations. Our contribution encompasses the design, synthesis, and characterization of a novel class of dicephalic-type cationic surfactants, branched on a methine motif, possessing two symmetric trimethylammonium groups, which constitute a double-head extension of the standard alkyltrimethylammonium salts of the single-head, single-tail structure. The convenient synthetic route and final purification steps allowed for the high-yield, high-purity production of the surfactants. Dicephalic-type surfactants demonstrated lower surface activity and higher critical micelle concentration values when compared with their single head–single tail counterparts. That can be attributed primarily to the presence of strong electrostatic repulsive forces within the bulky, double-charge headgroups and significant counterion condensation. Furthermore, molecular dynamics simulations demonstrated a propensity for the desorption of surfactants from the interface, even in diluted solutions, which constrained the attainable surface concentration and resulted in a lower reduction in surface tension. The mSTDE model of adsorption provided an excellent description of the experimental surface isotherms with a concise set of parameters. The model’s predictive power was demonstrated by the studies of the effect of inorganic salts on the surface activity of investigated surfactants. Our unique approach enabled us to gain a theoretical explanation of the newly devised surfactants’ behavior at the water/air interface.

## Introduction

The interest in designing highly specialized synthetic surfactants incorporating multicharged architecture has increased remarkably during the past few years.^1−6^ Dicephalic surfactants, having a single hydrophobic tail and two hydrophilic head groups,^7,8^ may constitute an outstanding group of amphiphiles with potential modern applications in chemical engineering and technology, as well as in biomedical fields.^9−14^ Those double-headed derivatives comprise, typically, a linking group with tertiary amine (e.g., iminodiacetic acid derivatives)^15^ or amide (e.g., *N*,*N*-bis[3,3′-(trimethylammonio)propyl]alkylamide salts)^7^ motifs. Additionally, in the case of multicharged surfactants, the formation of the specific transient complexes with their counterions was found to occur, causing the nonequivalent adsorption process at the liquid interfaces due to their effective charge decrease in relation to a nominal one.^9,10,16,17^

Dicephalic surfactants have received attention due to relatively easy synthetic routes and unique properties, especially in terms of interfacial behavior toward biomedical applications as stabilizers for drug/contrast agent carrier systems,^15,18,19^ and a linking group may behave as hydrophilic or hydrophobic, depending on the pH or ionic strength.^8,10,20,21^ Therefore, we designed and synthesized dicephalic-type cationic surfactants comprising exclusively carbon atoms, with an obvious exception for hydrophilic quaternary amine headgroups and counterions. In contrast to the aforementioned surfactants with linking motifs prone to undergo protonation/deprotonation,^7,15,18^ our newly devised compounds may be easily compared with alkyltrimethylammonium salts, playing the role of their single head–single tail analogues. Therefore, they can be regarded as the reference compounds for dicephalic cationic surfactants suitable for testing any theoretical approaches for the description of adsorption at fluid interfaces.

In our previous works concerning the adsorption of cationic surfactants and, in particular, dicephalic surfactants,^7,22^ we proposed the STDE adsorption model to describe their surface tension isotherms. The model was based on the Frumkin adsorption isotherm for surfactants and the assumption that surfactant counterions penetrate the Stern layer at the water interface, which can be regarded as a “surface quasi-two-dimensional electrolyte”, in which the electroneutrality condition was not satisfied.^23^ The model was successfully applied to describe the adsorption of the surfactants of various molecular structures. Moreover, using that model and the results of ionic concentration measurements, we demonstrated that the formation of surfactant-counterion associates needs to be considered for the dicephalic surfactants.^7,22^

Molecular dynamics (MD) simulations have emerged as a powerful tool, as they can capture the molecular structure and offer a complementary perspective to experimental studies. In MD simulations, the behavior of atoms and molecules is modeled computationally, allowing for a detailed exploration of interfacial structures and dynamics at the molecular level.^24−26^ For example, the MD simulations proved to be helpful in explaining the experimental observation in C*_n_*TAB-covered interfaces via changes in the interaction and organization,^27^ as well as revealing the molecular origin of synergistic effect in surfactant mixtures.^28,29^ In the case of multiheaded surfactants, MD has been mainly used to describe their aggregation behavior.^30,31^ All atom simulations, as well as those performed in coarse-grained resolution,^32^ demonstrated, for example, that with the increase in the number of charged head groups on the surfactants, the aggregation number decreases.

**Chart 1:**
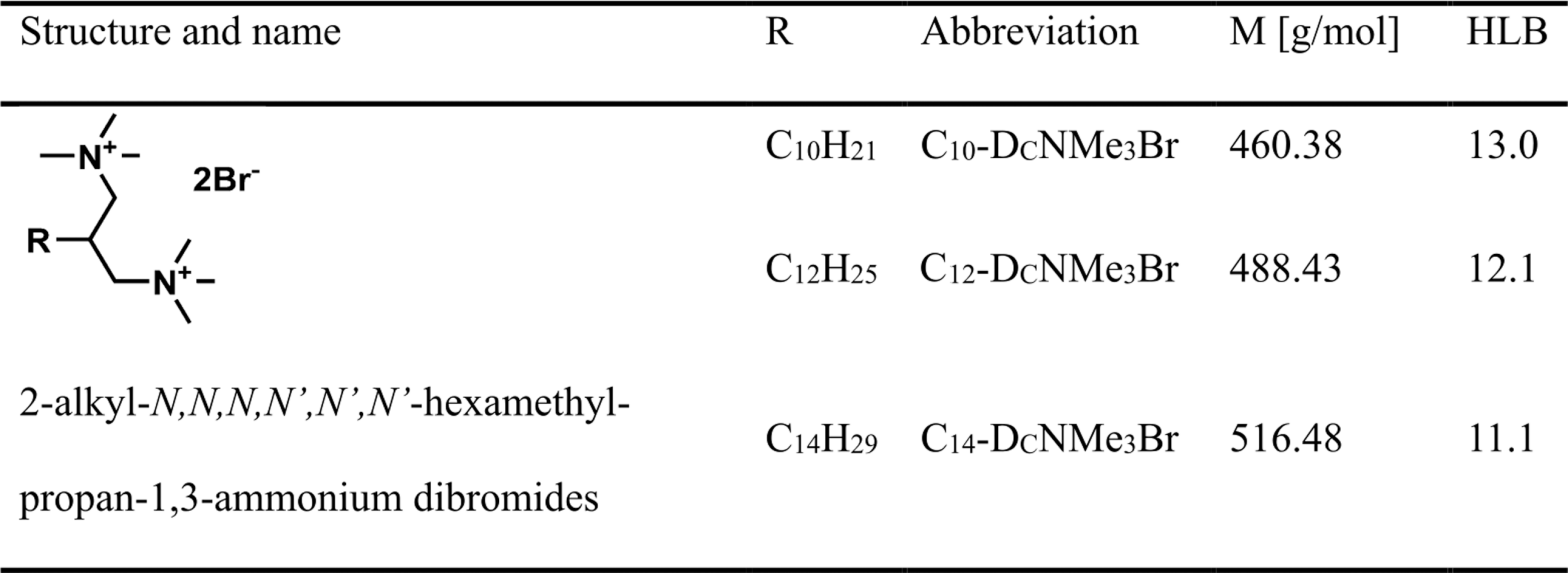
Designed Dicephalic Surfactants under Study

In this study, we focused on the design and synthesis of a set of dicephalic-type surfactants, 2-alkyl-*N,N,N,N*′*,N*′,*N*′-hexamethylpropan-1,3-ammonium dibromides (C_*n*_-D_C_NMe_3_Br), comprising exclusively carbon atoms in the hydrophobic part (see Chart 1, HLB values are calculated according to the McGowan method, and detailed information is shown in the Supporting Information).

We described the experimental isotherm of the designed dicephalic surfactants with a modified version of the STDE ionic surfactant model and confronted its results with those obtained by molecular dynamics simulations. That makes a unique combination of the synthesis of new surface-active compounds, experimental determination of their adsorption properties at the water/air interface, and their theoretical description by the thermodynamics-based model and MD simulations. Such a combination of methods can rarely be found in the literature.

## Materials and Methods

### Materials

All reagents, except organic solvents, were obtained from Merck. Solvents and inorganic salts (e.g., anhydrous sodium carbonate) were obtained from Avantor Performance Materials or Stanlab and used as received. The commercially available alkyltrimethylammonium bromide was purchased from Merck (dodecyl, DTABr) or from Carl Roth (tetradecyl, TTABr, and hexadecyl, CTABr). For the ^1^H NMR and ^13^C NMR analyses, surfactant samples were dissolved in DMSO-*d*_6_ (Merck) at concentrations between 5 and 10 mg/mL. Prior to FT-IR measurements, KBr (potassium bromide FT-IR grade, ≥99% trace metals basis, Merck) pellets with the analyzed compounds were prepared (press, 5–7 tons).

#### Synthesis of 2-Alkyl-*N,N,N,N*′*,N*′,*N*′-hexamethylpropan-1,3-ammonium Dibromides

The synthetic route for 2-alkyl-*N,N,N,N*′*,N*′,*N*′-hexamethylpropan-1,3-ammonium dibromides comprises four steps: alkylation of dimethyl malonate, reduction of the obtained intermediate, hydroxyl motif exchange into bromide ones, and final quaternization with trimethylamine. The alkylation step was performed in methanol for reagent molar ratio bromoalkane:dimethyl malonate:sodium equal to 1:1.05:1.05. After reaction completion (8 h of heating under reflux), products (dimethyl 2-alkylmalonates) were isolated by liquid–liquid extraction (water-diethyl ether) with yields of 95–99%. Dimethyl 2-alkylmalontes were reduced into appropriate 2-alkylpropane-1,3-diols using lithium aluminum hydride (50% excess) in tetrahydrofuran with yields of 78–83%, followed by further purification by crystallization from hexane. An exchange of hydroxyl groups into bromide motifs was performed in solventless conditions (heating at 130–140 °C, PBr_3_ with molar ratio to reactant 1:1), followed by the obtained 1-bromo-2-(bromomethyl)alkane isolation (liquid–liquid extraction, water:diethyl ether) with yields of 88–92%. The final quaternization step was performed in a pressure vessel using 430 mL of 30% trimethylamine in absolute ethanol and 430 mL of ethyl acetate per 1 mol of 1-bromo-2-(bromomethyl)alkane. After heating at 75–85 °C for 36 h, the reaction mixture was cooled down, followed by collecting of the precipitate – 2-alkyl-*N,N,N,N*′*,N*′,*N*′-hexamethylpropan-1,3-ammonium dibromides with yields of 73.5–86.7%.

#### Structure Characterization by Spectroscopic Techniques

^1^H NMR and ^13^C NMR spectra were recorded on a Bruker AMX-500 spectrometer, and chemical shifts (at 500 MHz for ^1^H NMR) were given in ppm. The obtained data were processed using Bruker TopSpin software (version 3.6.1). Mass spectra were determined by utilizing electrospray ionization mass spectroscopy (ESI-MS) (micrOTOF-Q instrument; Bruker Daltonics, Germany). The ESI-MS apparatus was operated in a positive ion mode (calibrated with the Tunemix mixture; Bruker Daltonics; Germany). The spectra were analyzed by DataAnalysis 3.4 software (Bruker Daltonics, Germany) with a resolution of at least 5 ppm. Fourier transform infrared (FT-IR) spectra were recorded on the Vertex 70 instrument (Bruker) using the KBr pellet method with a bandwidth of 4000–400 cm^–1^.

#### Melting Point Determination

Melting points were determined using the Boetius apparatus (Carl Zeiss, Jena, Germany), equipped with a heating table and a thermometer. The heating speed was equal to 4 °C/min for all of the samples. Each sample was measured in triplicate, and the average for measurements with a difference of less than 1 °C was taken as the result for a particular compound.

#### Elemental Analysis

Elemental analyses were performed utilizing the Vario EL cube (Elementar, Germany) calibrated on acetanilide and utilizing samples’ masses exceeding 100 mg.

#### HLB Calculations

HLB values were calculated using Davies and universal McGowan methods. Detailed information is provided in the Supporting Information.

#### Krafft Point Measurements

Krafft points of the studied surfactants were measured for 1% aqueous solutions according to the method described in ref (33) and supported by theoretical calculations.^34^ Samples’ aqueous solutions at a concentration of 1 wt % were prepared by dissolving the surfactant in Milli-Q water, followed by gentle agitation and heating when needed. When entirely dissolved at high temperatures, the solutions were cooled to induce precipitation, followed by an overnight equilibration. *T*_K_ was determined by gradual, controlled (1 °C/min) heating of 1 mL of the surfactant solution in a sealed test (NMR) tube until a clear solution was obtained. The acceptable reproducibility of three separate measurements was ± 0.1 °C. If no precipitate was found when the clear solutions were cooled to room temperature, the test tube was placed in an ice bath (equal to 0 °C) for 2 h. When no visible precipitation or turbidity was observed, the *T*_K_ of the sample solution was regarded as below 0 °C (<0 °C in Table 1). The group increment method for theoretical calculations of *T*_K_ is described in the Supporting Information.

**Table 1:** Structures, Chemical Characterization, and Properties of Dicephalic-Type Cationic Surfactants Branched on a Carbon Atom

		^1^H NMR, DMSO-*d*_6_	^13^C NMR, DMSO-*d*_6_		elemental analyses (theoretical values)							
abbreviation	molecular weight [g/mol]	δ [ppm]	δ [ppm]	ESI-MS (MNa^+^)	%C	%H	%N	MP [°C]	*T*_K_ [°C]	*T*_K_^a^ [°C]	HLB^b^	HLB^c^
C_10_-D_C_NMe_3_Br	460.38	0.84–0.87 [t, 3H, **CH**_**3**_(CH_2_)_7_]; 1.25–1.28 [m, 14H, CH_3_**(CH**_**2**_**)**_**7**_]; 1.40 [m, 2H, (CH_2_)_7_**CH**_**2**_CH_2_CH = ]; 1.50–1.52 [m, 2H, (CH_2_)_7_CH_2_**CH**_**2**_CH = ]; 2.75 [m, 1H, CH_2_CH_2_**CH**=]; 3.19 [s, 18H, CH[CH_2_N^+^**(CH**_**3**_**)**_**3**_]_2_]; 3.40–3.51 [m, 4H, CH[**CH**_**2**_N^+^(CH_3_)_3_]_2_]	14 [-(CH_2_)_9_**C**H_3_]; 20–35 [-(**C**H_2_)_9_CH_3_]; 21 [-**C**H = ]; 55 [-N^+^(**C**H_3_)_3_]; 72 [CH(**C**H_2_N^+^)_2_]	380.0	49.88 (49.57)	9.43 (9.65)	6.01 (6.09)	232–234	<0	–61.2	44.8	13.0
C_12_-D_C_NMe_3_Br	488.43	0.84–0.87 [t, 3H, **CH**_**3**_(CH_2_)_9_]; 1.25–1.27 [m, 18H, CH_3_**(CH**_**2**_**)**_**9**_]; 1.43 [m, 2H, (CH_2_)_9_**CH**_**2**_CH_2_CH = ]; 1.50–1.53 [m, 2H, (CH_2_)_7_CH_2_**CH**_**2**_CH = ]; 2.76 [m, 1H, CH_2_CH_2_**CH**=]; 3.23 [s, 18H, CH[CH_2_N^+^**(CH**_**3**_**)**_**3**_]_2_]; 3.43–3.58 [m, 4H, CH[**CH**_**2**_N^+^(CH_3_)_3_]_2_]	14 [-(CH_2_)_11_**C**H_3_]; 20–35 [-(**C**H_2_)_11_CH_3_]; 21 [-**C**H = ]; 55 [-N^+^(**C**H_3_)_3_]; 72 [CH(**C**H_2_N^+^)_2_]	408.3	51.97 (51.64)	10.10 (9.93)	5.64 (5.74)	228–229	<0	–48.4	43.9	12.1
C_14_-D_C_NMe_3_Br	516.48	0.84–0.87 [t, 3H, **CH**_**3**_(CH_2_)_11_]; 1.24–1.28 [m, 22H, CH_3_**(CH**_**2**_**)**_**11**_]; 1.42 [m, 2H, (CH_2_)_11_**CH**_**2**_CH_2_CH = ]; 1.48–1.50 [m, 2H, (CH_2_)_11_CH_2_**CH**_**2**_CH = ]; 2.74 [m, 1H, CH_2_CH_2_**CH**=]; 3.21 [s, 18H, CH[CH_2_N^+^**(CH**_**3**_**)**_**3**_]_2_]; 3.41–3.55 [m, 4H, CH[**CH**_**2**_N^+^(CH_3_)_3_]_2_]	14 [-(CH_2_)_13_**C**H_3_]; 20–35 [-(**C**H_2_)_13_CH_3_]; 21 [-**C**H = ]; 55 [-N^+^(**C**H_3_)_3_]; 72 [CH(**C**H_2_N^+^)_2_]	436.3	53.59 (53.48)	10.25 (10.17)	5.52 (5.43)	234–236	<0	–35.6	42.9	11.1

a Calculated according to the group increment method described in ref (34).

b Calculated according to Davies method.

c Calculated according to McGowan’s universal method.

#### Surface Tension Measurements

The analyzed surfactant solutions were prepared directly before measurements utilizing the dilution method (concentration of stock solution, mmol/mL). The equilibrium surface tension measurements were performed using the pendant drop shape analysis method with the DSA25 Expert Goniometer (Krüss, Germany), equipped with a Peltier-controlled temperature chamber, humidity control, and ADVANCE Software for data collection. Each solution, prepared around 1 h before studies, was measured at least in triplicate, and an average of results with a relative error of less than 1% were taken for surface tension determination by fitting to the Young–Laplace equation. All surface tension measurements were performed at 295 K and a relative humidity of 75–90%.

#### Molecular Dynamics Simulations

The Gromacs 2022.3 package,^35,36^ with the CHARMM^36,37^ force field, was used for all-atom molecular dynamics (MD) modeling of a selected dicephalic-type cationic surfactant branched on the carbon atom, that is, C_12_-D_C_NMe_3_Br, as well as its monovalent counterpart dodecyltrimethylammonium bromide (DTAB).The structure and topology of surfactants were generated using the CHARMM-GUI web server.^38–40^ For water, the four-point rigid water model (OPC4) was applied.^41^ It should be mentioned that the combination of the CHARMM force field with the OPC4 water model enables the accurate determination of surface tension.^42^

We performed MD simulations in bulk and on an air/water interface. We used the NPT ensemble for bulk simulation while for simulations at the interface NVT ensemble. Temperature coupling was controlled via V-rescale thermostat^43^ at a temperature of 298 K and coupling constant of 0.5 ps. The van der Waals interactions were described by the Lennard–Jones potential, smoothly shifted to zero between 1.0 and 1.2 nm. The electrostatic interactions were modeled by the PME method^44^ with a 1.2 nm cutoff, 0.12 nm grid spacing, and fourth-order splines. For simulations at the interface, we applied correction for the slab geometry.^45^ An adequate number of Br ions were added to make the simulation systems charge neutral in all simulations. The equations of motion were integrated using the leapfrog integration scheme and a 2 fs time step. Bonds involving hydrogen were constrained using LINCS^46^ and SETTLE^47^ algorithms. All molecular visualizations employ the VMD software package.^48^

For simulation in bulk, we studied surfactant solution at a concentration close to 1 × 10^2^ mol/dm^3^. In a cubic box with an initial size of 20.5 × 20.5 × 20.5 nm^3^, we inserted 48 surfactant molecules. After 200 steps of energy minimization, the system was simulated for 100 ns, from which the first 50 ns were considered as the initial equilibration period and disregarded from the analysis.

For the simulations at the liquid/gas interface, a system was a periodic rectangular simulation box, 8 × 8 × 24 nm^3^, consisting of a slab of water with a thickness of ∼8 nm and separated by a vacuum region. Initial configurations, generated using PACKMOL,^49^ were constructed by randomly placing surfactant molecules into two monolayers at opposite orientations. Surfactant headgroups were oriented toward the water slab, while the exact angle between the tail and the interface was chosen randomly. Amounts of the surfactant on the surface were chosen arbitrarily in a surface concentration range from Γ = 1.30 × 10^–7^ [mol/m^2^] to Γ = 3.25 × 10^–6^ [mol/m^2^] for both C_12_-D_C_NMe_3_Br and DTAB. For the simulations at the interface, we performed 500 steps of energy minimization and 350 ns long production run. Due to the long equilibration period at higher surfactant concentrations, only the last 100 ns were used for analysis.

The average surface tension was calculated from the difference between the normal and the lateral pressures using built-in Gromacs tools. The degree of counterion compensation in bulk simulations of C_12_-D_C_NMe_3_Br was calculated as a fraction of Br^–^ ions within the first coordination shell, that is, at a distance corresponding to the first minimum after the maximum of radial distribution function between N atoms in surfactant molecules and Br^–^ counterions. On the other hand, to calculate the surface charge compensation, we determined the number of Br^–^ counterions within the surfactant layer, that is, within the range corresponding to the surfactant peak on the mass density profile.

#### Adsorption Isotherm

In our previous works concerning the adsorption of cationic surfactants and, in particular, dicephalic surfactants,^7,22^ we proposed the STDE adsorption model to describe their surface tension isotherms. The model was based on the Frumkin adsorption isotherm for surfactant ions and the assumption that their counterions penetrate the Stern layer at the water interface, considered as a “surface quasi-two-dimensional electrolyte”, in which the electroneutrality condition was not satisfied.^23^ The model was successfully applied to describe the adsorption of surfactants of various molecular structures. Moreover, using that the model and the results of ionic concentration measurements, we demonstrated that for the dicephalic surfactants, the formation of surfactant-counterion associates needs to be considered.^7,22^

In the present paper, for the description of the adsorption of ionic surfactants supported by the MD simulations, we propose a modification of the STDE model (mSTDE) by using the Helfand–Frisch–Lebowitz (HFL) isotherm based on the equation of state of 2D hard disk-like particles.^50^ The equation for the 2D equation of state can also be derived based on the scaled particle theory.^51^ To account for the presence of counterions and surfactant-counterion associates in the Stern layer, we used the extended version mixtures of hard disks with various diameters^52^ in the mathematical form as follows:
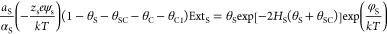
1for surfactant cations (S),
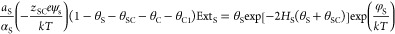
2

For surfactant-counterion associates (SC);
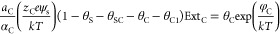
3
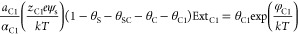
4for surfactant counterions (C) and anions of added electrolyte (C1), where
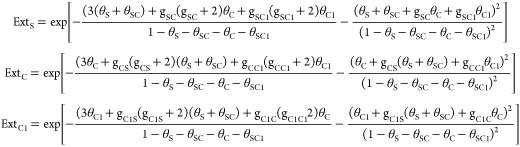
5and *a*_*i*_ = γ_*i*_*c*_*i*_ is the activity of the species *i*, *i* = (S, SC, C, C1) in the solution, γ_*i*_ and *c*_*i*_ are the respective activity coefficients, which can be computed using the extended Debye–Hückel theory^53^ and concentration; *z*_*i*_ is the valency of a given ion; α_S_ is the “surface activity” of the surfactant cation constituting a measure of the free energy of the adsorption after separating the contribution of the electric components, and α_C_ and α_C1_ are the “surface activities” of the counterions being a measure of their penetration into the Stern layer due to van der Waals interactions, image forces, and accounts for (partial) dehydration. For the multivalent ions, it also considers the correlation effects.  is the surface concentrations of a given species, while their limiting values are determined by the molecular geometry of the surfactant headgroups and counterions, , and *d*_*i*_ is the effective diameter of a given species, ; *H*_s_ is the interaction parameter accounting for the attractive lateral interactions among the adsorbed surfactant hydrophobic tails. Other parameters are defined similarly to those in ref (23). In particular, the electric potential of the Stern layer, ψ_s_, can be derived from
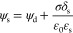
6

For the electrolyte with multivalent ions, no analytical formula relates the surface charge density:
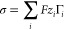
7and ψ_d_, the electric potential at the boundary between the Stern layer and the diffuse part of the EDL, so it needs to be calculated numerically using the implicit formula:
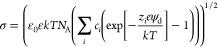
8where the contributions of all ionic species, that is, surfactant ions, their counterions, surfactant-counterion associates, and ions of added electrolyte with respective valencies *z*_*i*_, are included in the summation. Here, *F* and *N*_A_ are the Faraday constant and the Avogadro number, respectively, δ_s_ is the thickness of the Stern layer, ε_0_ is the vacuum permittivity, ε is the permittivity of an aqueous phase, and ε_s_ is the dielectric constant in the Stern layer. The activity corrections for the lateral interactions in a two-dimensional electrolyte φ_*i*_ can be determined by means of the formula:
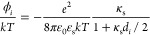
9where  is the two-dimensional analogue of the Debye–Hückel screening parameter.^54^ To evaluate the total surface excess concentrations of all ionic species, the adsorption in the diffuse part of the EDL has to be taken into account, namely:
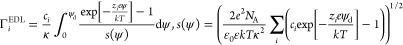
10and κ is the 3D Debye–Hückel reciprocal length. After the determination of the total surface excess concentrations of the surfactant ions and all other ions present in the solution, the surface tension can be derived from integrating the Gibbs adsorption equation:
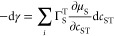
11where  is the total surface excess concentrations (including that in the diffuse part of the EDL) for surfactant cations (S), surfactant-counterion associates (SC), surfactant counterions (C), and anions (C1) and cations (K1) of the added salt, μ′s, μ_*i*_ = μ_*i*0_ + RT ln(*a_i_*), are the respective chemical potentials, where μ_*i*0_ denotes the standard chemical potential and *c*_ST_is the total concentration of surfactant (including associates) in the solution.

Similarly, as in our previous works concerning the adsorption of dicephalic surfactants, we consider the formation of surfactant-counterion associates in terms of the mass action law:
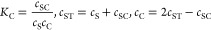
12

An additional equation based on the mass action law for the salt anions needs to be considered in the presence of the added salt.

## Results and Discussion

### Design, Synthesis, and Properties of the Studied Surfactants

The design of the studied surfactants involves a unique dicephalic-type structure, comprising exclusively carbon atoms in all structural elements (hydrophobic tail, branching motifs, etc.), with an exception for hydrophilic headgroups and counterions. Such structures are extremely interesting due to their unique properties, especially multiplied headgroups, and lack of other chemical motifs prone to protonation/deprotonation. Considering the structures of the designed surfactants, it was evident that any direct synthetic route, like the alkylation of a hydrophilic headgroup-bearing fragment, cannot be utilized with satisfactory yields and purities of the final products. Thus, it was necessary to utilize an appropriate multistep synthetic route involving the introduction of one chemical motif into a hydrophobic fragment followed by its conversion into the final product. Our approach for dicephalic cationic surfactants involved the alkylation of dimethyl malonate with an appropriate alkyl bromide, followed by intermediate reduction to the diol and exchange of hydroxyl groups for bromide motifs. The latter derivative was quaternized using an excess of trimethylamine to yield 2-alkyl-*N,N,N,N*′*,N*′*, N*′-hexamethylpropan-1,3-ammonium dibromides, our final products. Taking into account the methods reported in the literature^55,56^ for the preparation of 2-alkylmalonates and 2-alkylpropane-1,3-diols followed by conversion into dibromides, we designed appropriate routes for a series of final products with a decyl, dodecyl, and hexadecyl alkyl chain.

For the alkylation step, a round-bottom flask equipped with a reflux condenser, magnetic stirrer, and dropping funnel was filled with 400 mL of methanol, and then, 19.36 g (0.84 mol) of metallic sodium was carefully added. The mixture was cooled in order to maintain a temperature of around 50 °C. Next, 110.97 g (0.84 mol) of dimethyl malonate was dropwise added under intensive stirring. Heating was continued for 15 min, followed by a dropwise addition of 0.8 mol of appropriate bromoalkane: 1-bromodecane (C_10_–Br, 176.94 g), 1-bromododecane (C_12_–Br, 199.38 g), or 1-bromotetradecane (C_14_–Br, 221.82 g). The reacting mixture was heated under reflux for 8 h, followed by evaporation of methanol up to the total volume of the mixture of ca. 300–350 mL. The concentrated reaction mixture was treated with 150 mL of distilled water. The organic layer was separated while the aqueous one was extracted in triplicate with diethyl ether (3 × 30 mL). The combined organic phases were dried over anhydrous Na_2_SO_4_, followed by diethyl ether evaporation under reduced pressure. Yields: 95% (dimethyl 2-decylmalonate, C_10_-D_C_COOMe, 207.01 g), 97% (dimethyl 2-dodecylmalonate, C_12_-D_C_COOMe, 233.14 g), 99% (dimethyl 2-tetradecylmalonate, C_14_-D_C_COOMe, 260.16 g).

The reduction of the obtained derivatives was conducted in a round-bottom flask equipped with a reflux condenser, a magnetic stirrer, and a dropping funnel. Next, 600 mL of tetrahydrofuran was introduced, followed by the careful addition of 37.95 g (1 mol) of lithium aluminum hydride. Subsequently, 0.67 mol of the appropriate dimethyl 2-alkylmalonate, dissolved in 150 mL of tetrahydrofuran, was added dropwise to a vigorously stirred solution of lithium aluminum hydride over 60 min. Following the addition of dimethyl 2-alkylmalonate, the mixture was maintained under reflux for 3 h. The warm reaction mixture was then carefully poured into 1000 mL of 10% hydrochloric acid under conditions of vigorous stirring. Once the mixture had cooled to room temperature, it was extracted with three portions of dichloromethane (3 × 250 mL). The organic layers were then combined, dried over anhydrous MgSO_4_, and evaporated to dryness in vacuo. Yields of crude products: 78% (2-decylpropane-1,3-diol, C_10_-D_C_OH, 113.07 g), 83% (2-dodecylpropane-1,3-diol, C_12_-D_C_OH, 135.92 g), 81% (2-tetradecylpropane-1,3-diol, C_14_-D_C_OH, 147.87 g). Crude products were purified by crystallization from hexane (500 mL), filtration, and drying in vacuo at room temperature.

For an exchange of hydroxyl groups into bromide motifs, a set of a round-bottom flask equipped with a reflux condenser connected to a dropping funnel, magnetic stirrer, and capillary for the introduction of an inert gas was utilized. An appropriate 2-alkylpropane-1,3-diol (0.50 mol) was introduced, followed by careful heating and stirring until its complete liquefaction. Then, the temperature was carefully elevated to 130–140 °C, during continuous stirring and flushing with dry nitrogen, and maintained for 24 h. After reaction completion, the mixture was cooled to room temperature and treated with 150 mL of diethyl ether and 50 mL of cold water. The mixture was transferred to a separating funnel, followed by addition of 15 mL of 30% hydrogen peroxide until the aqueous phase was completely colorless. The organic layer was separated, while the aqueous one was extracted with diethyl ether (2 × 50 mL). Combined organic layers were dried over anhydrous Na_2_SO_4_ and evaporated to dryness in vacuo. Yields of crude products were 88% (1-bromo-2-(bromomethyl)dodecane, C_10_-D_C_Br, 150.55 g), 91% (1-bromo-2-(bromomethyl)tetradecane, C_12_-D_C_Br, 168.44 g), and 92% (1-bromo-2-(bromomethyl)hexadecane, C_14_-D_C_Br, 183.20 g). Those crude products were used for further synthesis without purification, although (if needed) they were distilled under reduced pressure.

In order to synthesize dicephalic cationic surfactants, an appropriate 1-bromo-2-(bromomethyl)alkane (0.35 mol) was placed in a pressure vessel with 150 mL of a 30% solution of trimethylamine in absolute ethanol and 150 mL of ethyl acetate. The mixture was heated to 75–85 °C for 36 h and then placed in a refrigerator (temperature: 0–5 °C) for 24 h. After that, the mixture was filtered, and the precipitate was dried in vacuo for 24 h. Yields of crude products were 73.5% (2-decyl-**N*,*N,N,N*′*,N*′*,N*′*-hexamethylpropan-1,3-ammonium dibromide, C_10_-D_C_NMe_3_Br, 118.43 g), 79.1% (2-dodecyl-*N*,*N,N,N*′*,N*′*,N*′-hexamethylpropan-1,3-ammonium dibromide, C_12_-D_C_NMe_3_Br, 135.22 g), and 86.7% (2-tetradecyl-**N*,*N,N,N*′*,N*′*,N*′*-hexamethylpropan-1,3-ammonium dibromide, C_14_-D_C_NMe_3_Br, 156.72 g).

The first two steps, alkylation of dimethyl malonate and reduction of the obtained intermediate, are well established for synthesizing 2-alkylpropane-1,3-diols. Typically, for long-chain alkyl bromides, the methods involve the use of an excess of malonate (up to 1.5 equiv) and an appropriate base (sodium methoxide/ethoxide or NaOH; up to 1.4 equiv), and the reaction is carried out in a polar solvent (preferably low molecular weight alcohol or *N*,*N*-dimethylformamide).^55,56^ Such synthetic procedures assume that long alkyl chain bromide reacts completely and thus should not produce any impurity. The obtained 2-alkylmalone is isolated by extraction and may be further purified by vacuum distillation. The yield for derivatives with longer (dodecyl) alkyl chain is higher than with the shorter (decyl) one: 92% versus 80%. To complete, the obtained 2-alkylmalone may be converted by reduction, preferably with an excess of LiAlH_4_ (1.1 to 1.25 equiv). The crude products are isolated by extraction and purified by crystallization from ethyl acetate^55^ or hexane.^56^ Yields for the dodecane derivative (86%) are also higher when compared with 2-decylpropane-1,3-diol (77%). The third step, conversion of 2-alkylpropane-1,3-diols into 1-bromo-2-(bromomethyl) alkanes, could be, preferably, performed in solventless conditions, utilizing an excess (1.5 equiv) of phosphorus tribromide as a brominating agent. The isolated, by extraction from the water–diethyl ether system, products may be further purified by distillation under reduced pressure. Literature yields for dodecyl derivative is 82.5%.^56^

**Scheme 1:**
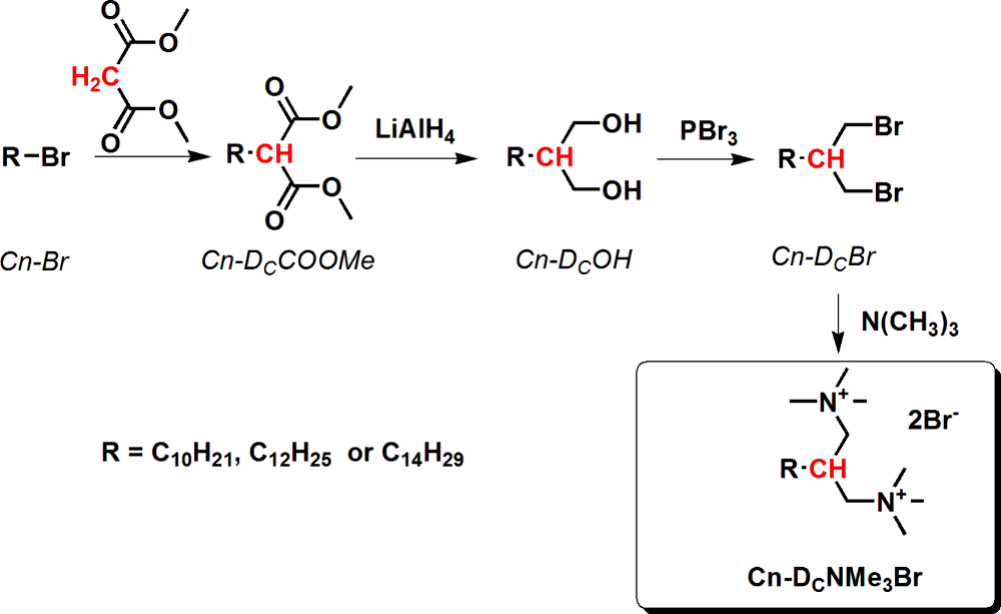
General Synthetic Routes for the Studied Dicephalic Surfactants (C_*n*_-D_C_NMe_3_Br)

The entire synthetic route of the C_*n*_-D_C_NMe_3_Br surfactants is shown in Scheme 1. The first three steps of the synthesis were designed and performed on the basis of the literature procedures,^55,56^ with some modifications. In order to convert the obtained C_*n*_-D_C_Br derivatives to cationic dicephalic surfactants, we designed a novel synthetic method. The original, last step, resulting in the desired 2-alkyl-**N*,*N,N,N*′*,N*′*,N*′*-hexamethylpropan-1,3-ammonium dibromide, was performed under conditions (ethanol–ethyl acetate mixture as a solvent system, excess of trimethylamine as a reagent), which enable precipitation of the formed product, thus facilitating isolation and purification.

The synthetic routes devised enabled us to obtain the final dicephalic-type surfactants in good yields and purities. The ester derivatives, see C_*n*_-D_C_COOMe, were obtained with yields 95–99%, and their purities, assessed by ^1^H NMR, were sufficiently good, so the distillation step was unnecessary. Moreover, dimethyl 2-tetradecylmalonate, was solid (mp 45–46 °C), so it was possible to purify it by crystallization from hexane. It should be noted that our yields were significantly higher when compared with the literature ones (80% for decyl derivative and 92% for dodecyl one), most possibly due to diethyl malonate exchange for dimethyl malonate (lower boiling point, possibility to use of more violate methanol as the solvent) and lack of necessity to use additional vacuum distillation.^55^ The yields for 2-alkylpropane-1,3-diols were very similar to the literature ones: 78% (lit. 77%) for C_10_-D_C_OH and 83% (lit. 86%) for C_12_-D_C_OH.^55^ It is worth noticing that compounds with shorter (decyl) chains are generally obtained with lower yields than those with longer (dodecyl) chains, most possibly due to higher aqueous solubilities for both C_10_-D_C_COOMe and C_10_-D_C_OH, resulting in higher losses during extraction. Dibromides, C_*n*_-D_C_Br, were also obtained with high yields (88–92%), exceeding the literature one (82.5% for C_12_-D_C_Br),^56^ and purities (confirmed by ^1^H and ^13^C NMR), enabling their further use without vacuum distillation.

Chemical characterization of the C_*n*_-D_C_NMe_3_Br surfactants is given in Table 1, while appropriate ^1^H and ^13^C NMR, as well as FT-IR spectra, are provided in the Supporting Information (Figures S1 and S2). It should be noted that distillation under reduced pressure of ester (C_*n*_-D_C_COOMe) and dibromide (C_*n*_-D_C_Br) derivatives may result in their partial degradation and thus lower yields and even purities. So, that step should be omitted, if possible. The final surfactants, C_*n*_-D_C_NMe_3_Br, were obtained by quaternization of C_*n*_-D_C_Br dibromides in a closed vessel, utilizing alcoholic trimethylamine (33%) mixed with ethyl acetate as a quaternizing agent and solvent system. Such an approach enabled the use of both an elevated (75–85 °C) temperature at the first stage and cooling afterward to induce crystallization from the reaction mixture.

It should be noted that crude products, that is, obtained just by the filtration of the precipitated material, followed by washing it with a few portions of ethyl acetate, were characterized by sharp melting points (turned liquid within a maximal range of 2 °C) and NMR (both ^1^H and ^13^C) spectra comprising no significant signals attributed to impurities. The characteristic peaks, demonstrated in Figure S1, with the lowest chemical shift value at c.a. 0.85 ppm and c.a. 14 ppm for ^1^H NMR and ^13^C NMR, respectively, were observed for the methyl group at the end of the hydrophobic alkyl chain. It should be noted that this signal was utilized for calibration of the integrated intensities for ^1^H NMR spectra (relative values of three protons). Signals of methylene groups in the alkyl chain, neighboring only with aliphatic carbon atoms, appeared as signals at 1.2–1.5 ppm (^1^H NMR) and 20–35 ppm (^13^H NMR). The methine motif was observed for chemical shifts of ca. 2.75 ppm (^1^H NMR) and 21 ppm (^13^H NMR). Signals with the highest values of chemical shifts (ca. 3.21 ppm and 3.40–3.60 ppm for ^1^H NMR as well as 55 and 72 ppm for ^13^H NMR) were attributed to methyl and methylene groups (relative integrated intensities for ^1^H NMR are 9:2) in hydrophilic headgroups, respectively. The spectra confirmed the presence of duplicated trimethylammonium groups attached to a single hydrophobic tail with a terminal methyl motif. The lack of any signals in ^13^H NMR with chemical shifts >80 ppm indicated that there were no unreacted carbonyl motifs or degradation products with double carbon–carbon bonds in the final product.

FT-IR spectra of our new surfactants, reported in Figure S2a in the Supporting Information, revealed the presence of sharp signals at 2850–3015 and at 1375–1485 cm^–1^, attributed to C–H stretching and C–H bending/scissoring, respectively. The lack of strong signals at 1650–1750 cm^–1^ confirms the presence of no carbonyl motifs from the impurities. The spectra are in good agreement with the theoretical one obtained for the C_12_-D_C_NMe_3_ cation using the DFT computations for a single molecule (Figure S2b).

Appropriate structures and purities of the obtained compounds were also confirmed by ESI-MS and elemental analyses, the experimental values were in good agreement with theoretical ones (for any element: C, N, and H, the relative error was less than 2%).

### Validation of the mSTDE Model

**Figure 1:**
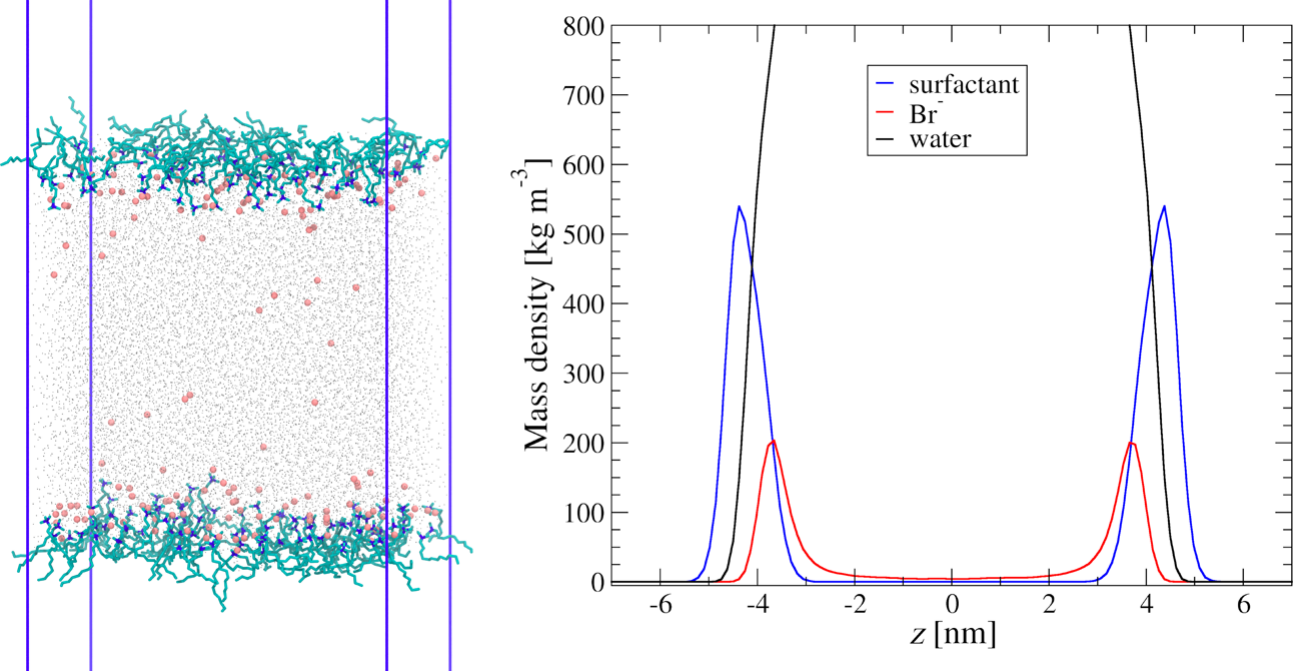
Left: snapshot from the MD simulations of the DTAB adsorption at the air/water interface for surface concentration Γ = 1.94 × 10^–6^ [mol/m^2^]; right: density profiles across the simulation slab for DTA cations, Br anions, and water.

For the validation of the modified STDE model of ionic surfactant adsorption, we measured the surface tension of single-head cationic surfactants dodecyltrimethylamonium bromide (DTAB), tetradecyltrimethylammonium (TTAB), and cetyltrimethylammonium bromide (CTAB). We also performed MD simulations for DTAB to model the structure of the interfacial layer at the water/air interface and extract some mSTDE model parameters from their results. The example of the snapshots of the simulation for surface concentration Γ = 1.94 × 10^–6^ mol/m^2^ is illustrated in Figure 1, together with resulting density profiles in the simulation slab for water, surfactant molecules, and bromide counterions. Figures illustrating snapshots and density profiles for other surface concentrations are presented in the Supporting Information (Figures S3 and S4).

The results of the MD simulations shown in Figure 1 corroborate the assumption of the modified STDE model that the counterions penetrate the interfacial layer where the cationic headgroups of surfactants are located and, thus, partly compensate the surface charge due to their adsorption.

**Figure 2:**
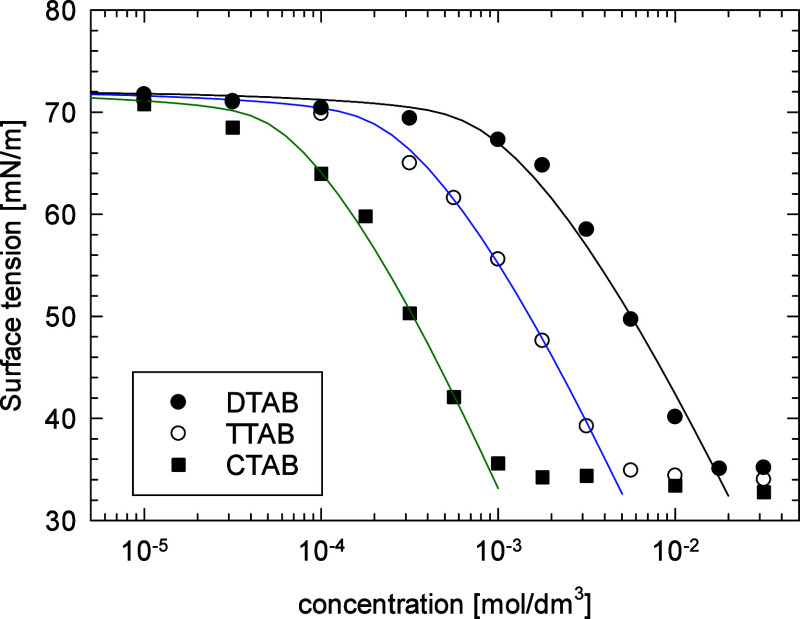
Surface tension isotherm of DTAB, TTAB, and CTAB. Points: experimental results; lines: best fit to the modified STDE model of ionic surfactant adsorption.

The experimental results for the surface tension isotherms for DTAB, TTAB, and CTAB are illustrated in Figure 2, together with the best-fit lines of the modified STDE model to experimental points. They agree well with the ones described previously.^23^ The best-fit model parameters for the surfactant cations are collected in Table 2. The values of surface activity α_S_ and interaction parameter *H*_s_are close to ones for nonionic surfactants *n*-alkyl phosphine oxides with the same hydrocarbon chain length,^57^ while the effective size of the CTA^+^ head was close to the molecular size determined for the optimized structure used for the MD simulation. Other model parameters, bromide surface activity α_Br_, and the dielectric constant in the Stern layer δ_s_ were the same as in ref (23). The effective size of the bromide anion was close to the nonhydrated molecular size (0.36 nm),^58^ and the thickness of the Stern layer was taken from the MD simulations (c.f. Figure 1.).

**Table 2:** Best Fit Parameters for the Modified STDE Model

surfactant	α_s_[mol/dm^3^]	a_s_ [nm]	H_s_ [kJ/mol]	χ^2^ [(mN/m)^2^]
DTAB	1.8 × 10^–5^	0.42	6.0	1.70
TTAB	1.8 × 10^–6^	0.42	8.0	0.67
CTAB	1.2 × 10^–7^	0.42	10.0	1.81

**Figure 3:**
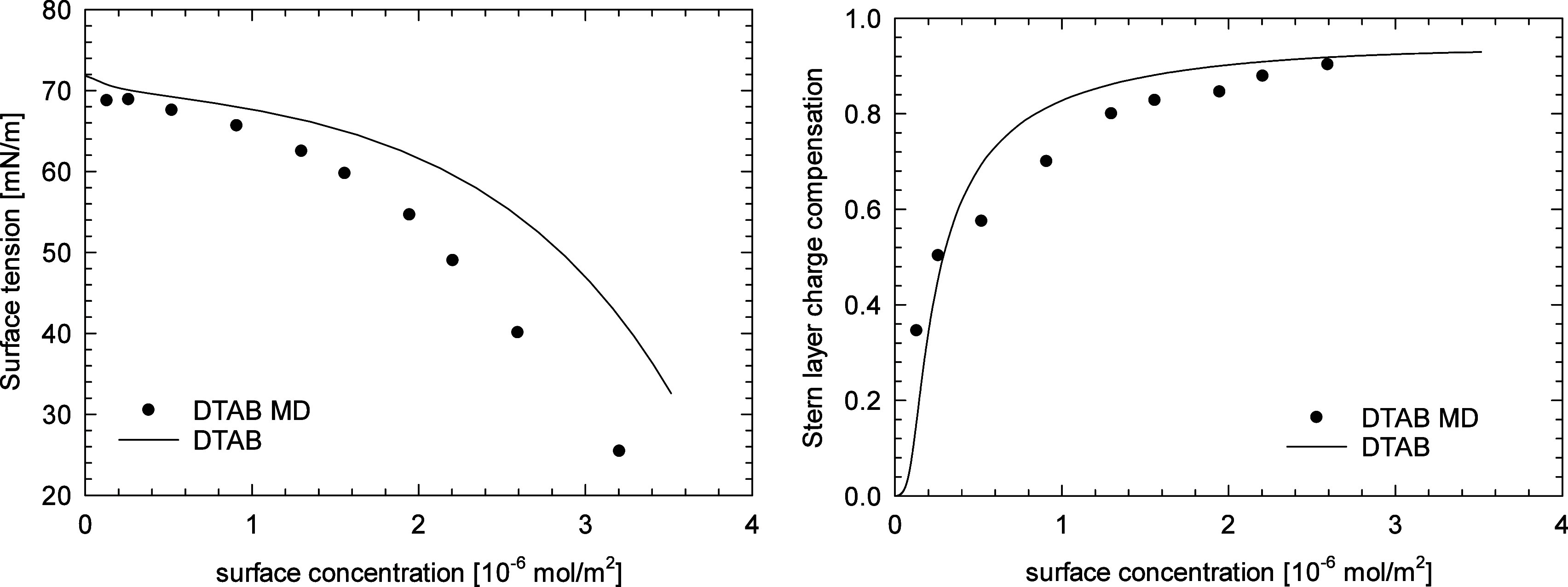
Comparison of the results of MD simulations derived from the modified STDE model fitted to the experimental data for DTAB surface tension. Left: surface tension vs surface concentration; right: Stern layer charge compensation degree vs surface concentration.

Figure 3 presents the comparison of the dependency of surface tension and the degree of headgroup charge compensation in the Stern layer by bromide anions on the surfactant surface concentration derived from the MD simulations and calculated from the best fit of the mSTDE model to the experimental data. One can observe the qualitative agreement between the results of MD simulations and the thermodynamic model applied to describe the experimental surface tension isotherm. It seems that the MD simulations tend to overestimate the effect of surfactant on the surface tension, which can be attributed to the specific force field used, that is, overestimation of the electrostatic repulsions between the surfactant molecules, size of the simulation box, uncertainty of the surfactant bulk concentration in the slab, or the limited time accessible for the simulations. The origin of the observed discrepancy between the MD simulations and the mSTDE model will be investigated in the near future.

### Adsorption of Dicephalic Surfactants at the Air/Water Interface

**Figure 4:**
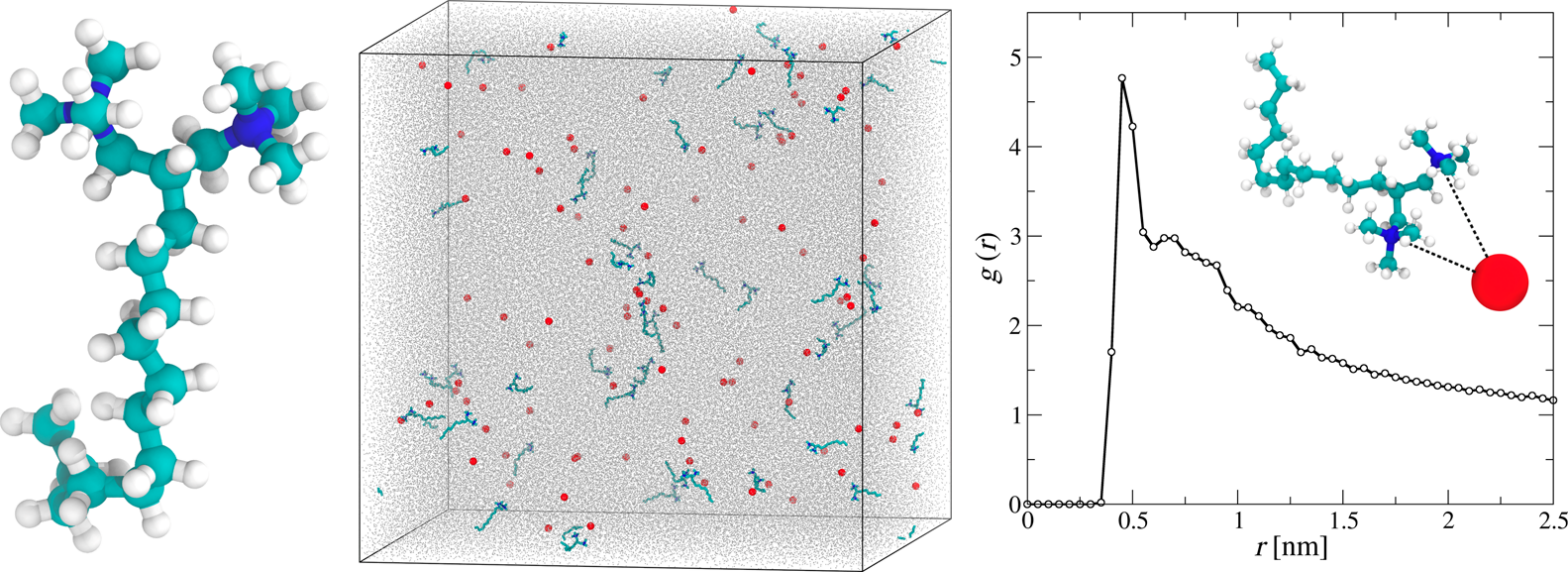
On the left is the surfactant molecular structure. In the middle, the snapshot of the MD configurations after 100 ns production run corresponding to C_12_-D_C_NMe_3_Br at a concentration of ∼1 × 10^–2^ M determined from MD simulations. Black lines represent the periodic boundaries of the simulations box. Br^–^ ions are presented as red spheres, while water’s O atoms are drawn as gray points. On the right, the radial distribution function between N atoms in surfactant molecules and Br^–^ counterions, as shown in the inset.

To provide an additional molecular-scale insight into the properties and behavior of newly synthesized surfactant molecules, the solutions of C_12_-D_C_NMe_3_Br in bulk and on the gas/water interface were investigated via MD simulations. Figure 4 shows the snapshot of the 100 ns long MD simulations of surfactant solution at ∼1 × 10^–2^ M concentration. As observed at that concentration, the surfactant molecules do not aggregate. It is in agreement with the experimental results as the critical micelle concentration (CMC) of the surfactant has been determined as 3 × 10^–2^ M (see below). That can be related to their significant charge, which, on the other hand, is compensated for by the Br^–^ counterions. This was quantitatively investigated by the radial distribution function (RDF), between the surfactant charge groups and the counterions (see Figure 4), exhibiting a maximum at a distance of ∼0.5 nm. That confirms the hypothesis on the presence of surfactant-counterion associates in the bulk solution. That affects overall surfactant adsorption at the air/water interface as the associates exhibit lower electrostatic repulsion of the positive surface charge than nonassociated surfactant molecules. For the 1 × 10^–2^ M C_12_-D_C_NMe_3_Br concentration, the degree of condensation was close to 10%.

**Figure 5:**
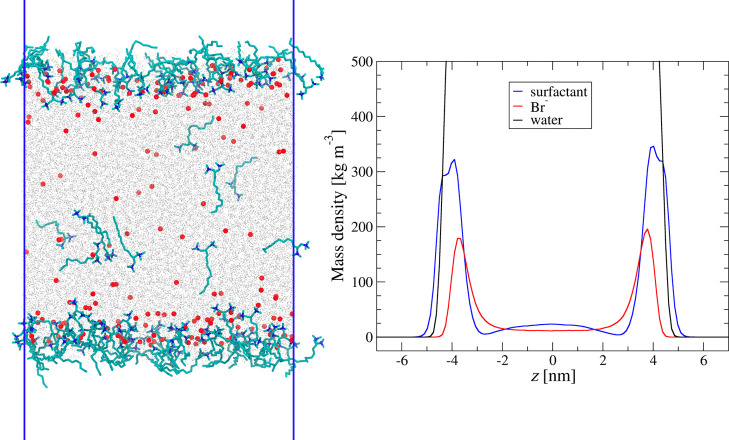
Left: snapshot from the MD simulations of the C_12_-D_C_NMe_3_Br adsorption at the air/water interface for surface concentration Γ = 1.16 × 10^–6^ [mol/m^2^]; right: density profiles across the simulation slab for C_12_-D_C_NMe_3_ cations, Br anions, and water.

Figure 5 illustrates the example of the snapshots of the simulation of the C_12_-D_C_NMe_3_Br adsorption at the water/air interface for surface concentration Γ = 1.94 × 10^–6^ mol/m^2^, together with resulting density profiles in the simulation slab for water, surfactant molecules, and bromide counterions. Figures illustrating snapshots and density for other surface concentrations are presented in the Supporting Information (Figure S5). The results of simulations presented in Figure 5 suggest a high degree of surface charge compensation by bromide counterions that penetrate the interfacial Stern layer. Interestingly, we observed that the surfactant molecules, even for relatively low surface concentrations (∼0.7 × 10^–6^ mol/m^2^), desorb from the interface and remain in bulk, forming premicellar aggregates. That is quantitatively shown in the mass density profiles in Figure S3b in the Supporting Information. Such behavior can be explained by strong electrostatic repulsions between the surfactant headgroups, as well as their relatively large size, which hinders the organization of the hydrophobic tails. Despite the surfactant desorption, the surface tension decreases with an increasing number of surfactant molecules, attaining a value of 45 mN/m for the highest surface concentration investigated.

The experimental results for the surface tension isotherm for C_10_-D_C_NMe_3_Br, C_12_-D_C_NMe_3_Br, and C_14_-D_C_NMe_3_Br are illustrated in Figure 2, together with the best-fit lines of the modified STDE model to experimental points. The best-fit model parameters for surfactant cations are given in Table 3. The effective size of the C_12_-D_C_NMe_3_ headgroup was chosen close to the molecular size determined for the optimized structure used for the MD simulation (geometrical average of main diameters of the headgroup, 0.59 nm). Other model parameters, such as bromide anion surface activity, their effective diameter, and the dielectric constant in the Stern layer, were assumed to be the same as for TAB model surfactants. The thickness of the Stern layer (δ_s_ = 1.7 nm) was chosen from the MD simulations (c.f. Figure 5.).

**Table 3:** Best Fit Parameters for the Modified STDE Model

surfactant	α_s_ [mol/dm^3^]	*a*_s_ [nm]	*H*_s_ [kJ/mol]	*K*_Br_ [dm^3^/mol]	χ^2^ [(mN/m)^2^]
C_10_-D_C_NMe_3_Br	2.0 × 10^–4^	0.56	7.0	10	0.53
C_12_-D_C_NMe_3_Br	1.9 × 10^–5^	0.56	9.5	10	0.55
C_14_-D_C_NMe_3_Br	1.8 × 10^–6^	0.56	12.0	10	1.61

**Figure 6:**
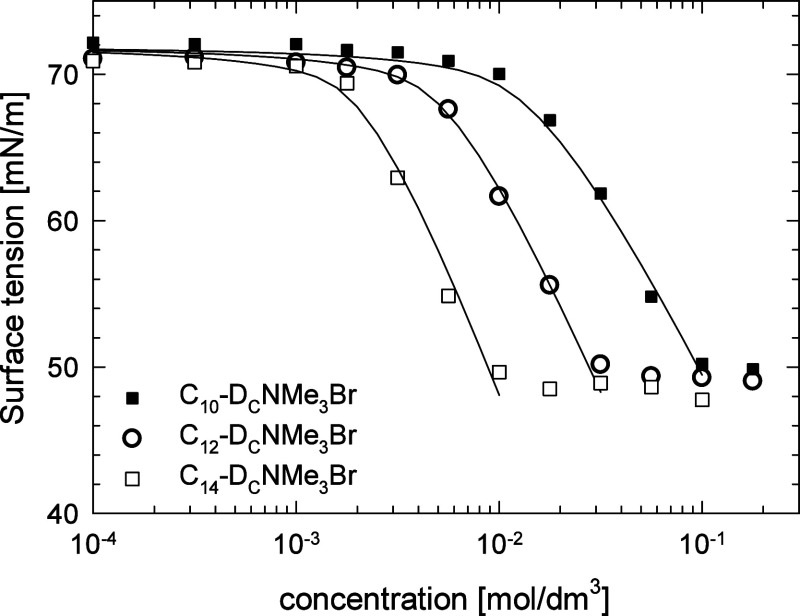
Surface tension isotherm of C_10_-D_C_NMe_3_Br, C_12_-D_C_NMe_3_Br, and C_14_-D_C_NMe_3_Br. Points: experimental results; lines: best fit to the modified STDE model of ionic surfactant adsorption.

The dicephalic C_*n*_-D_C_NMe_3_Br is characterized by a lower surface activity and higher CMC value than their single-head counterparts due to stronger electrostatic repulsion of the surfactant by the charge in the adsorbed layer. However, the repulsion is diminished when surfactant-counterion associates are formed. That effect can be effectively reproduced by the mSTDE adsorption model, as illustrated in Figure 6. The surface activity parameter, α_s_, was the same as for TABs with the same length of the hydrocarbon chain that governs the magnitude of the hydrophobic effect for a given surfactant molecule and represents the characteristic dependence on the number of carbon atoms in the chain. On the other hand, the value of surface tension at CMC (ca. 48 mN/m) for C_*n*_-D_C_NMe_3_Br is considerably higher than for their single-head counterparts (ca. 35 mN/m), which can be attributed to the more bulky highly charged headgroup and strong counterion condensation. That result is consistent with MD simulations, which showed that high surface coverage cannot be attained. The value of the equilibrium constant *K*_Br_ = 10 dm^3^/mol indicates that at CMC = 0.03 mol/dm^3^, 30% of surfactant molecules are in the form of associates.

**Figure 7:**
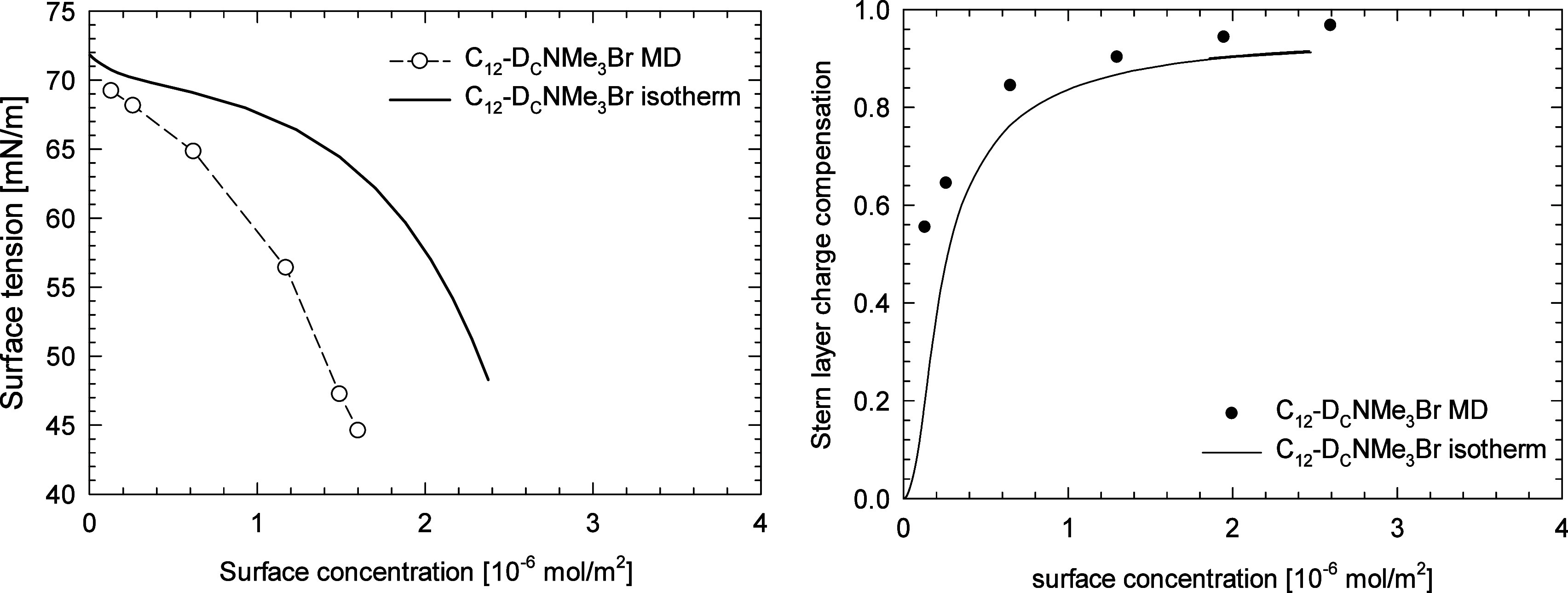
Comparison of the results of MD simulations and derived from the modified STDE model fitted to the experimental data for C_12_-D_C_NMe_3_Br surface tension; left: surface tension vs surface concentration; right: Stern layer charge compensation degree vs surface concentration.

Similarly as for DTAB, the MD simulations for C_12_-D_C_NMe_3_Br overestimate the surface activity. As shown in Figure 7, the surface concentration vs surface tension relationship derived from the model description of the experimental isotherm resulted in much higher surface tension values. The degree of charge compensation in the Stern layer, although in qualitative agreement (cf. Figure 7), seems to be overestimated when compared with the isotherm-derived values, although the electrolyte model used for MD simulations did not account for the ion polarizability or image forces.^59^ As the charge compensation is concentration dependent, the observed overestimation can be attributed to the limited size of the MD simulation box in the *z* direction, resulting in a higher concentration of Br^–^ ions than that in the experiments.

**Figure 8:**
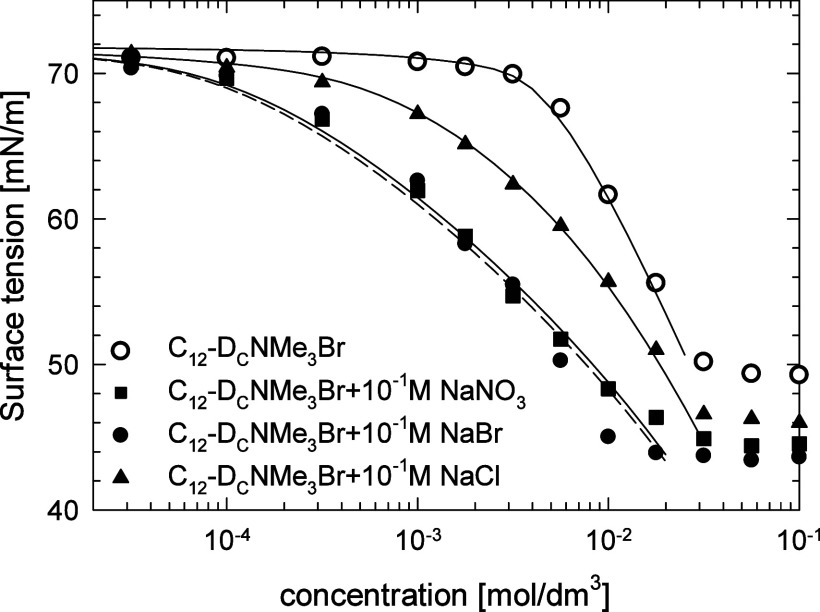
Surface tension isotherm of C_12_-D_C_NMe_3_Br in water and with the addition of 10^–1^ M salts, NaBr, NaCl and NaNO_3_. Points: experimental results; lines: best fit to the modified STDE model of ionic surfactant adsorption.

To investigate the effect of added salts on the surface tension of dicephalic surfactant C_12_-D_C_NMe_3_Br, we performed its measurements in the presence of 0.01 and 0.1 M NaBr, NaCl, and NaNO_3_. While the addition of 0.01 M of salt had a negligible effect on surface tension, 0.1 M of added salt had a considerable lowering effect. Similarly, as observed in our previous works, the effect depended on the added salt type of anion.^7,23^ As shown in Figure 8, the decrease in the surface tension in the presence of chloride anions was much lower than for bromide or nitride, and the effect of the latter was similar. The addition of salt caused an increase in the surfactant surface activity and decreased the surface tension at CMC, which is typical for ionic surfactants due to screening of the electrostatic repulsion. The mSTDE model of ionic surfactants’ adsorption well described the experimental results with the counterion parameters collected in Table 4 and defined previously for TAB surfactants.^23^ See Figure S6 for the description in terms of the mSDTE model of the surface tension of CTAB in the presence of those salts. The anion association constants were as following: *K*_Br_ = 10 dm^3^/mol, *K*_Cl_ = 7 dm^3^/mol, and  = 10 dm^3^/mol. No fitting procedure was used to obtain good agreement with the experimental isotherms in the presence of salts.

**Table 4:** Model Parameters for Electrolytes

parameter	value
ε_s_	24
δ_s_	1.5 nm
*d*_Br_	0.32 nm
α_Br_	2800 mol/dm^3^
*d*_Cl_	0.31 nm
α_Cl_	17000 mol/dm^3^
	0.31 nm
	2500 mol/dm^3^

## Conclusions

The unique class of dicephalic type cationic surfactants, characterized by a pH insensitive branching motif (methine) as well as the presence of exclusively hydrocarbon-borne structure (with the exception of positive charge bearing group and counterion), as an extension of classical alkyltrimethylammonium salts, was synthesized and studied. We devised not only the convenient synthetic route but also the necessary purification steps to obtain surfactants of sufficiently high purity for the determination of their physicochemical properties.

For the description of the surface tension isotherms of the dicephalic cationic surfactants, we developed the modified “surface quasi-two-dimensional electrolyte” (mSTDE) model of ionic surfactant adsorption. It uses the Helfand–Frisch–Lebowitz (HFL) isotherm to describe the adsorption of surfactant cations and considers counterion penetration into the interfacial Stern layer. That assumption, as well as the one on the formation of surfactant-counterion associates in the case of cationic dicephalic surfactants, was confirmed by the molecular dynamics simulations of surfactant solutions in bulk and at the interface. Some of the mSTDE model parameters could be established by MD calculations, which reduced the number of free parameters obtained by fitting the experimental results.

The adsorption properties of our newly devised surfactants were compared with their linear single head, single tail structure analogues, alkyltrimethylammonium salts, when alkyl is dodecyl, tetradecyl, and hexadecyl (cetyl). The mSTDE model description fitted to the experimental results was confronted with the results of the molecular dynamics simulations, with the conclusion that the MD overestimates the decrease of the surface tension at a given surfactant surface concentration. That was observed for both single-head, single-tail compounds and our dicephalic-type cationic surfactants. It is most probably due to the overrating of electrostatic interactions in the interfacial layer. The lower surface activity and the higher CMC value of our newly devised surfactants, when compared with their single-head analogues, may be attributed to stronger electrostatic repulsion by the charge of the adsorbed layer as well as the presence of bulkier, highly charged headgroups and strong counterion condensation. Moreover, in the MD simulations, we observed the desorption of the surfactant molecules from the interface into the bulk phase, even for relatively low surface concentration, due to strong electrostatic repulsive forces between the surfactant headgroups alongside their relatively large size, hindering the organization of the hydrophobic tails. That correlated with the observed higher surface tension value at CMC for the dicephalic surfactants compared with their single-head analogues.

The reduction of the electrostatic repulsion in the surface layer was evidenced by surface tension measurements in the presence of inorganic salts, showing the good predictive power of the mSTDE model. It followed the previously observed trend that the decrease of the surface tension in the presence of chloride anions was much lower than that in bromide or nitride, and the effect of the latter was similar.

Our newly designed and synthesized dicephalic-type cationic surfactants possess great potential for both theoretical studies and practical applications due to their distinctive structure and absence of pH-sensitive fragments. The most important factor affecting their interfacial adsorption and aggregation constitutes the presence of strong repulsive forces between bulky headgroups and significant counterion condensation and neutralization of the surface layer.

## Supporting Information Available

Theoretical determination of Krafft points; the calculation of HLB by Davies and McGowan methods; ^1^H NMR and ^13^C NMR spectra of C_*n*_-D_C_NMe_3_Br derivatives; FT-IR spectra of C_*n*_-D_C_NMe_3_Br derivatives; molecular dynamics simulations of DTAB and C_12_-D_C_NMe_3_Br; surface tension isotherms of CTAB in the presence of salts; application of the mSTDE model of ionic surfactant adsorption (PDF)
