# Supplementary material for: Synthesis of New Cationic Dicephalic Surfactants and Their Nonequivalent Adsorption at the Air/Solution Interface
Source: Langmuir. 2025 Mar 21;41(12):8125–37. doi: 10.1021/acs.langmuir.4c04803 (PMC11966777; doi:10.1021/acs.langmuir.4c04803)
Supplement: Supplementary file 1 — la4c04803_si_001.pdf [file la4c04803_si_001.pdf]

SUPPLEMENTARY MATERIAL

Synthesis of New Cationic Dicephalic Surfactants and Their Nonequivalent Adsorption at the Air/Solution Interface

Łukasz Lamch[*a], Izabella Leszczyńska[b], Daria Długowska[a], Weronika Szczęsna - Górniak[a], Piotr Batys[b], Ewelina Jarek[b], Kazimiera Anna Wilk[a], Piotr Warszyński[*b]

[a)] Department of Organic and Pharmaceutical Technology, Faculty of Chemistry, Wrocław University of Science and Technology, Wybrzeże Wyspiańskiego 27, 50-370 Wrocław, Poland

[b)] Jerzy Haber Institute of Catalysis and Surface Chemistry, Polish Academy of Sciences, Niezapominajek 8, 30-239 Kraków, Poland

* Corresponding authors

E-mail addresses:

lukasz.lamch@pwr.edu.pl (Ł. Lamch) and piotr.warszynski@ikifp.edu.pl (P. Warszyński).

1. The theoretical determination of Krafft points

In order to enable T_K of novel surfactants, an appropriate group increment method has been developed and described in[1]. Briefly, a statistical analysis of 227 anionic, 46 cationic, and 27 zwitterionic surfactants enabled us to distinguish appropriate chemical groups and their additive increments of positive or negative values into the total T_K of the whole surfactant molecule. T_K value comprises an algebraic sum of all chemical groups' increments (for repeating fragments, their number is multiplied by their single increment). The appropriate data and calculations are given below:

chemical group	$-CH_2-$	$-CH_3$	-CH<	$-N^+Me_3$	T_K
increment	6.415 °C	-15.521 °C	1.681 °C	-55.768 °C	
$C10D_cNMe_3Br$	11	1	1	2	-61.226 °C
$C12D_cNMe_3Br$	13	1	1	2	-48.396 °C
$C14D_cNMe_3Br$	15	1	1	2	-35.566 °C

2. The calculation of HLB by the Davies method

HLB value by Davies method comprises the use of the group increment method. HLB value comprises an algebraic sum of all chemical groups' increments (for repeating fragments, their number is multiplied by their single increment). The appropriate data and calculations are given below:

chemical group	$-CH_2-$	$-CH_3$	-CH<	$-N^+Me_3$	HLB
increment	-0.475	-0.475	-0.475	22	
$C10D_cNMe_3Br$	11	1	1	2	44.825
$C12D_cNMe_3Br$	13	1	1	2	43.875
$C14D_cNMe_3Br$	15	1	1	2	42.925

3. The calculation of HLB by the universal McGowan method

The most universal theory of HLB is provided by McGowan and Sowada. It enables the calculation of the HLB of any given compound: such value may be negative or positive, although 7 is the equilibrium – compounds with HLB values exceeding 7 are considered to be hydrophobic. The general formula is given by the equation:

$$HLB_M = 7 - 0.00337V_x + 1.5n_b$$

Where V_x denotes total molar volume, while n_b is the number of water molecules involved in the hydration of the molecule. In order to simplify the method, a dozen approximations have been introduced, enabling the calculation of CMC values as the simple sum of the group increments:

chemical group	$-CH_2-$	$-CH_3$	-CH<	$-N^+Me_3$	Br^- (counterion correction)	n_i (hydration number)	HLB
increment	-0.475	-0.658	-0.292	6.984	-0.88	6	
$C10D_cNMe_3Br$	1	11	1	2	2	12	13.033
$C12D_cNMe_3Br$	1	13	1	2	2	12	12.083
$C14D_cNMe_3Br$	1	15	1	2	2	12	11.133

4. ^1H NMR and ^{13}C NMR spectra of $C_nD_CNMe_3Br$ derivatives

^1H NMR and ^{13}C NMR spectra of $C_nD_CNMe_3Br$ derivatives with assignments of particular chemical motifs.

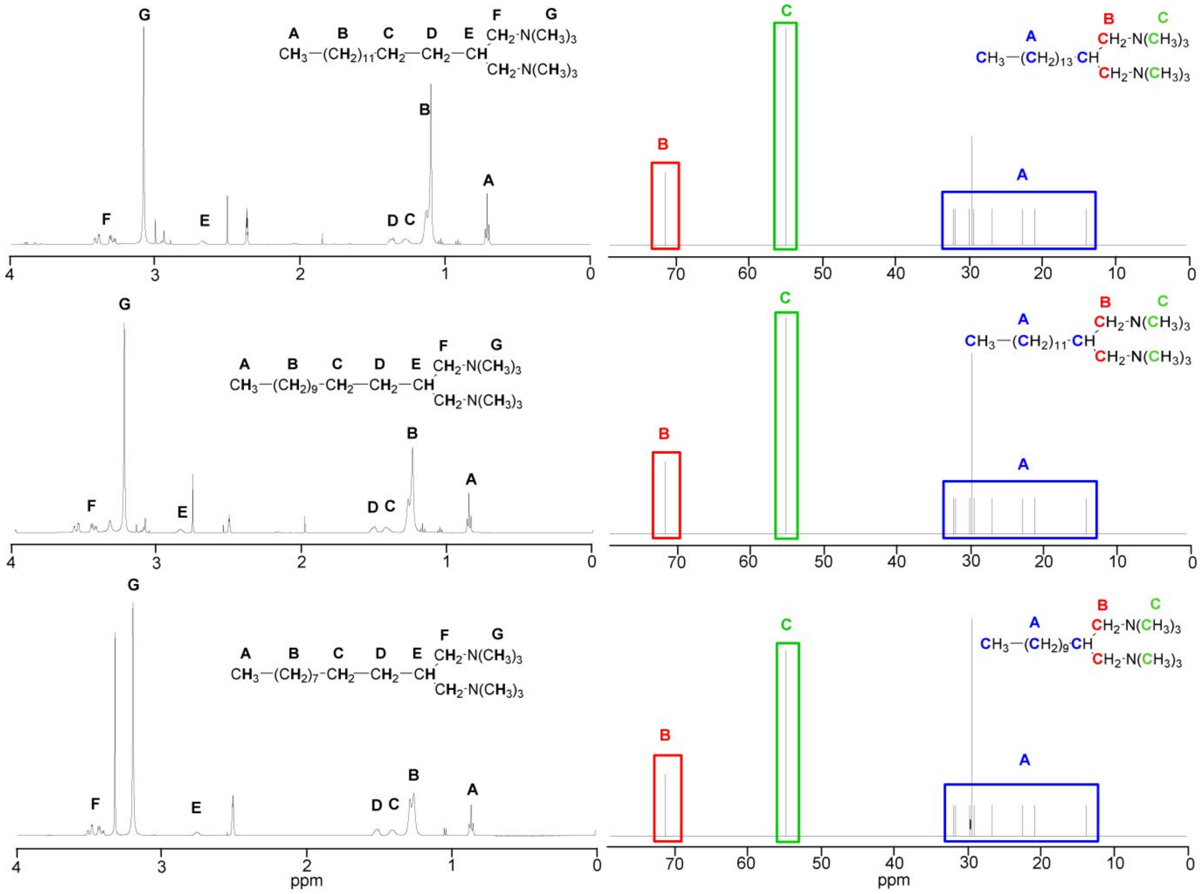

Fig. S1. ^1H and ^{13}C NMR spectra of $C_{10}D_CNMe_3Br$ (bottom), $C_{12}D_CNMe_3Br$ (middle) and $C_{14}D_CNMe_3Br$ (up).

5. FT-IR spectra of $C_nD_CNMe_3Br$ derivatives

FT-IR spectra of $C_nD_CNMe_3Br$ derivatives.

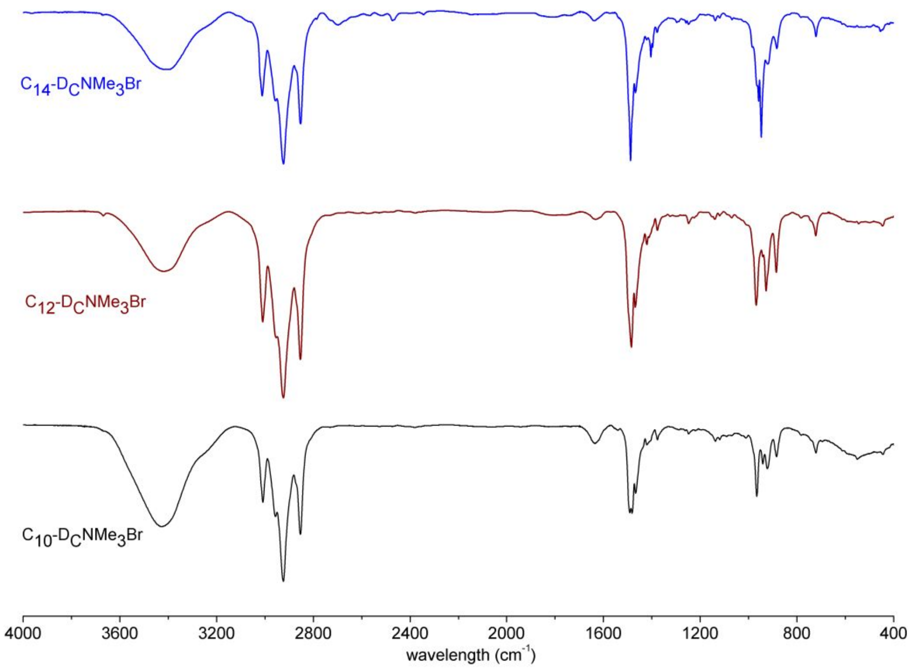

Fig. S2a. FT-IR spectra of $C_{10}D_CNMe_3Br$ (bottom), $C_{12}D_CNMe_3Br$ (middle) and $C_{14}D_CNMe_3Br$ (up).

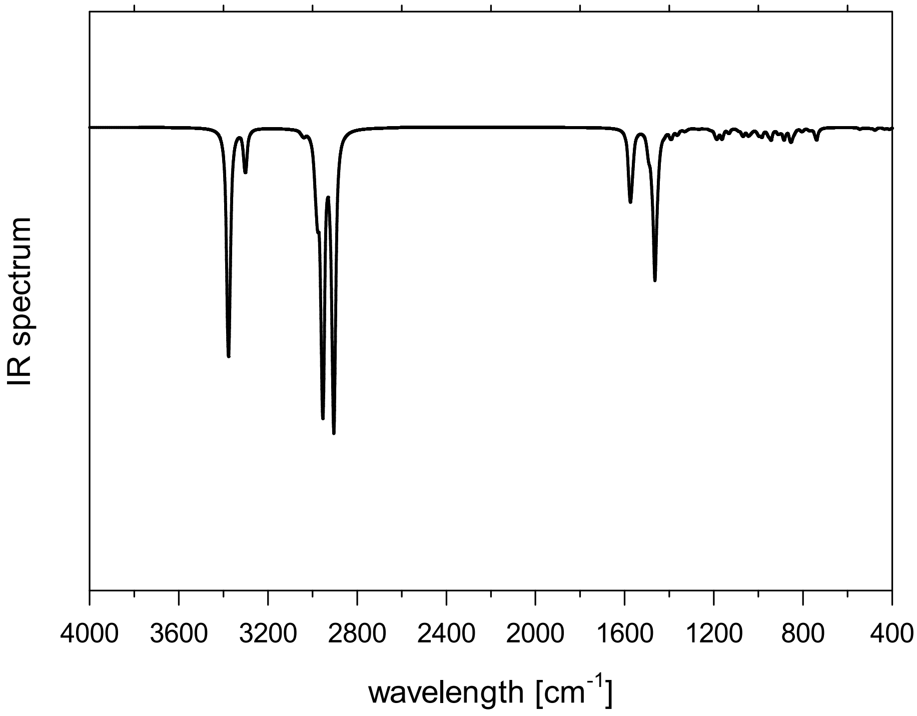

Fig. S2b. Single-molecule IR spectrum for $C_{12}D_CNMe_3$ cation calculated with the Gaussian 16 software using DFT method with wb97xD functional, 6-311G+(d,p) basis set [2]. The resulting IR spectra were rescaled according to [3].

6. Molecular Dynamics simulations of DTAB

Snapshots from the simulations of DTAB for various surface concentrations after a run of 100 ns are given below.

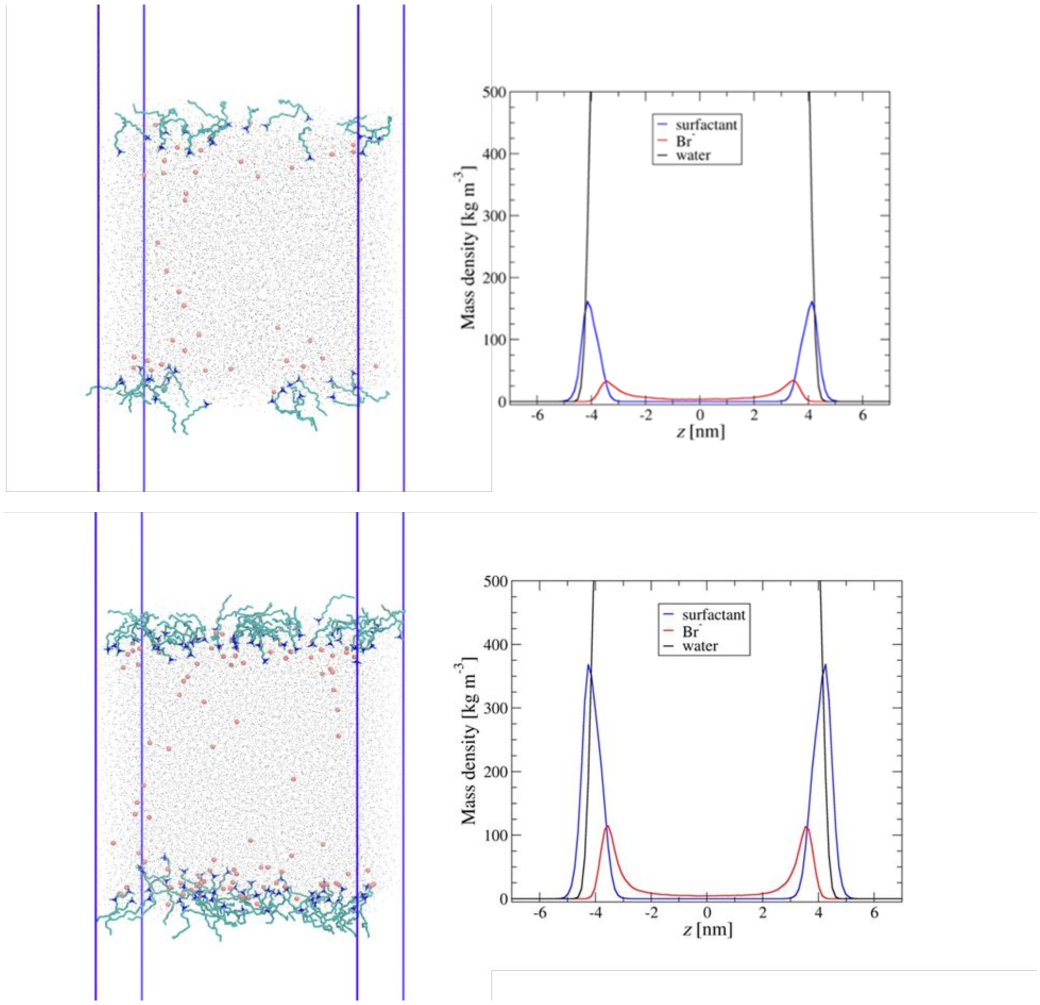

Fig. S3a. Snapshots showing surfactant configurations on gas/water interface after 100 ns of MD simulations. On the right, the mass density distribution across the *z*-direction. Surface concentrations: upper - 0.52×10^{-6} mol/m^3 , lower -1.3×10^{-6} mol/m^3.

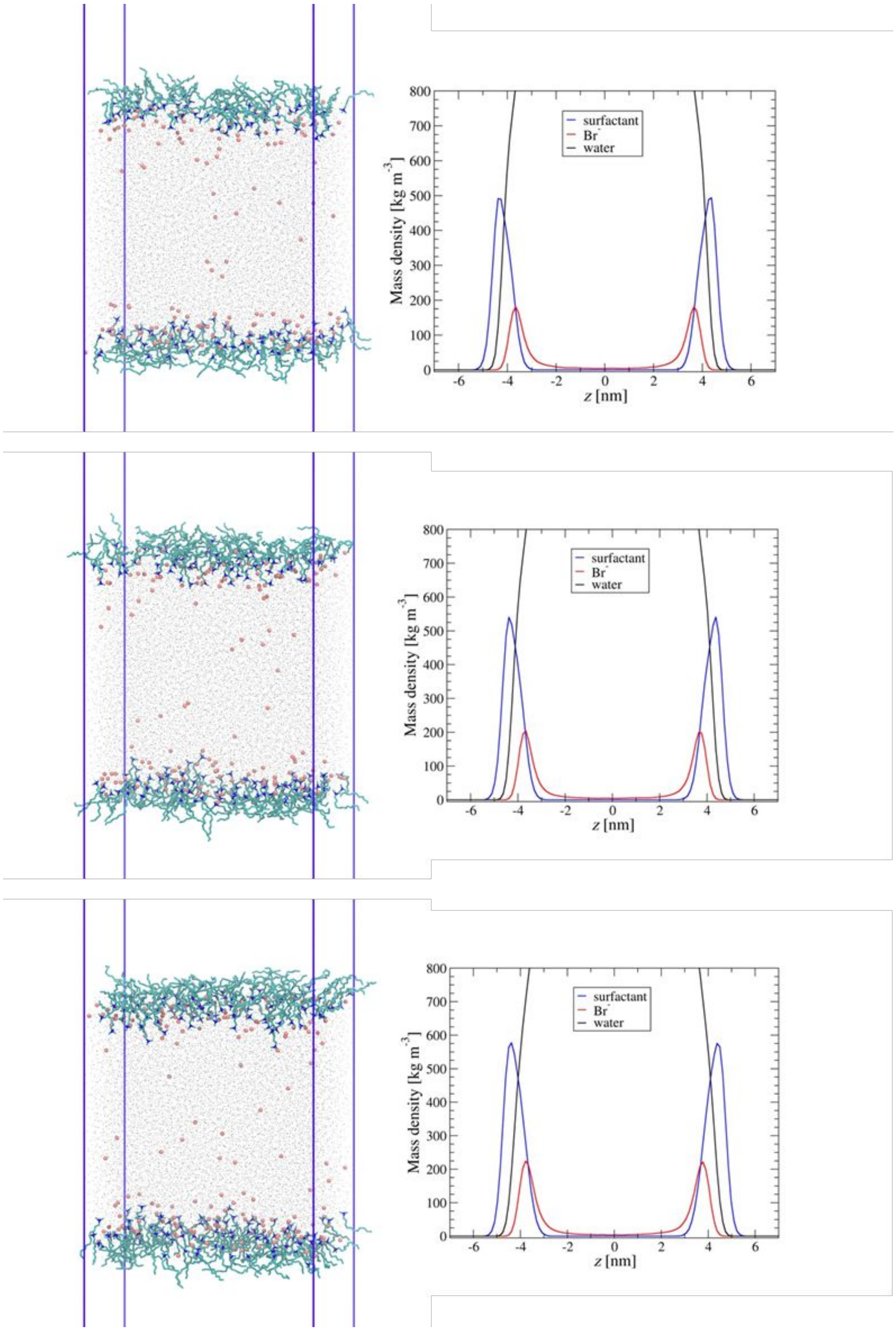

Fig. S3b. Snapshots showing surfactant configurations on gas/water interface after 100 ns of MD simulations. On the right, the mass density distribution across the *z*-direction. Surface concentrations: upper - 1.95×10^{-6} mol/m^3, middle - 2.2×10^{-6} mol/m^3 lower - 2.6×10^{-6} mol/m^3.

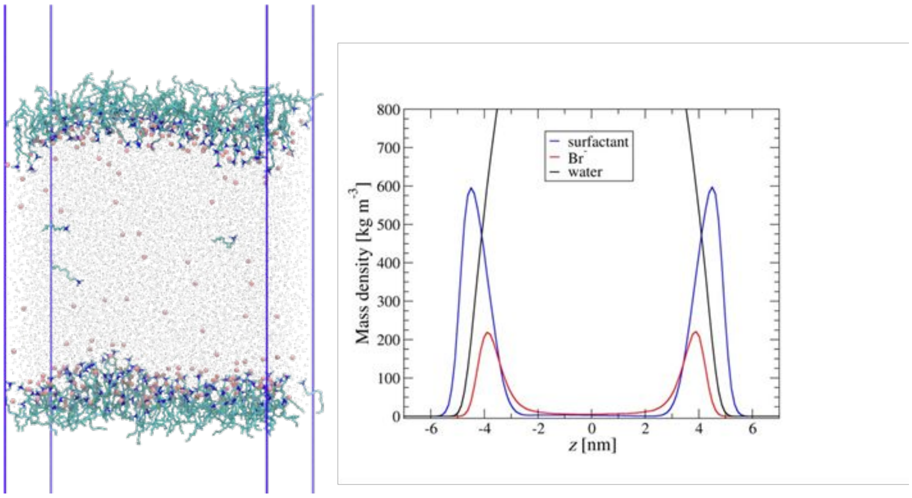

Fig. S3c. Snapshots showing surfactant configurations on gas/water interface after 100 ns of MD simulations. On the right, the mass density distribution across the *z*-direction. Surface concentration 3.3×10^{-6} mol/m^3.

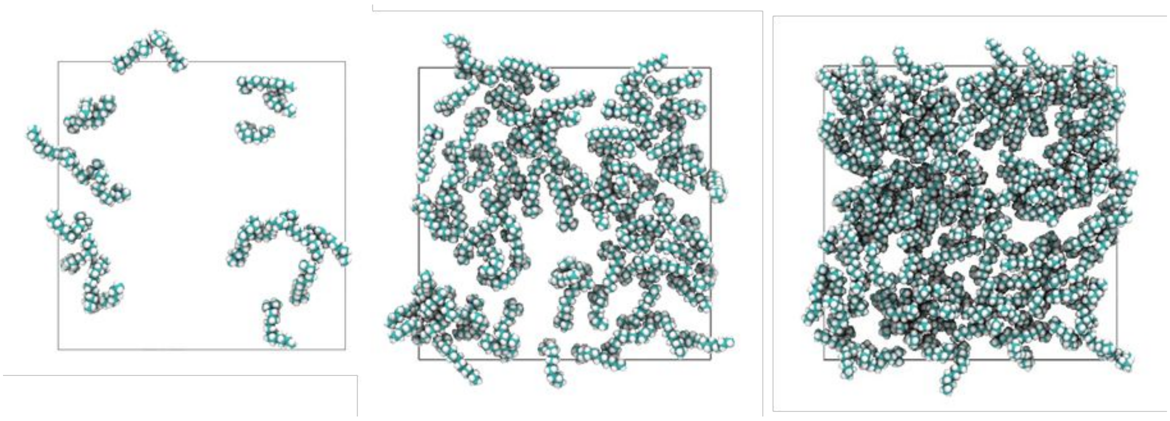

Fig S4. Simulation snapshots illustrating surface arrangement of adsorbed DTA^+ surface active cations in the van der Waals spheres representation for surface concentrations: left - 0.52×10^{-6} mol/m^3, middle - 1.94×10^{-6} mol/m^3, and 3.2×10^{-6} mol/m^3.

7. Molecular Dynamics simulations of C_{12}-$D_C NMe_3Br$

Snapshots from the simulations of C_{12}-$D_C NMe_3Br$ for various surface concentrations after a run of 350 ns are given below.

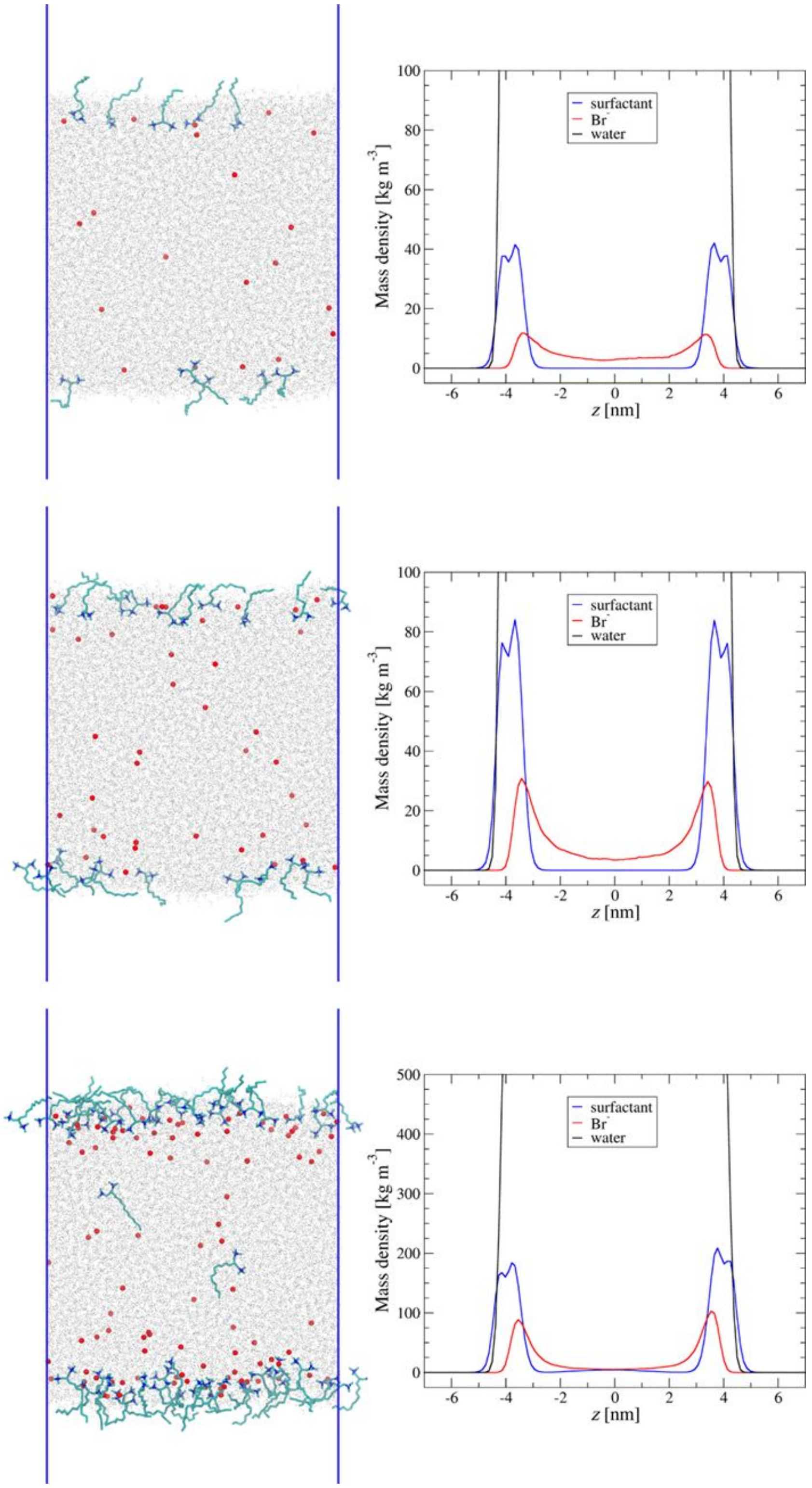

Fig. S5a. Snapshots showing surfactant configurations on gas/water interface after 350 ns of MD simulations. On the right, the mass density distribution across the *z*-direction. From top to bottom: surface concentrations 0.13×10^{-6} mol/m^3, 0.26×10^{-6} mol/m^3, and 0.62×10^{-6} mol/m^3.

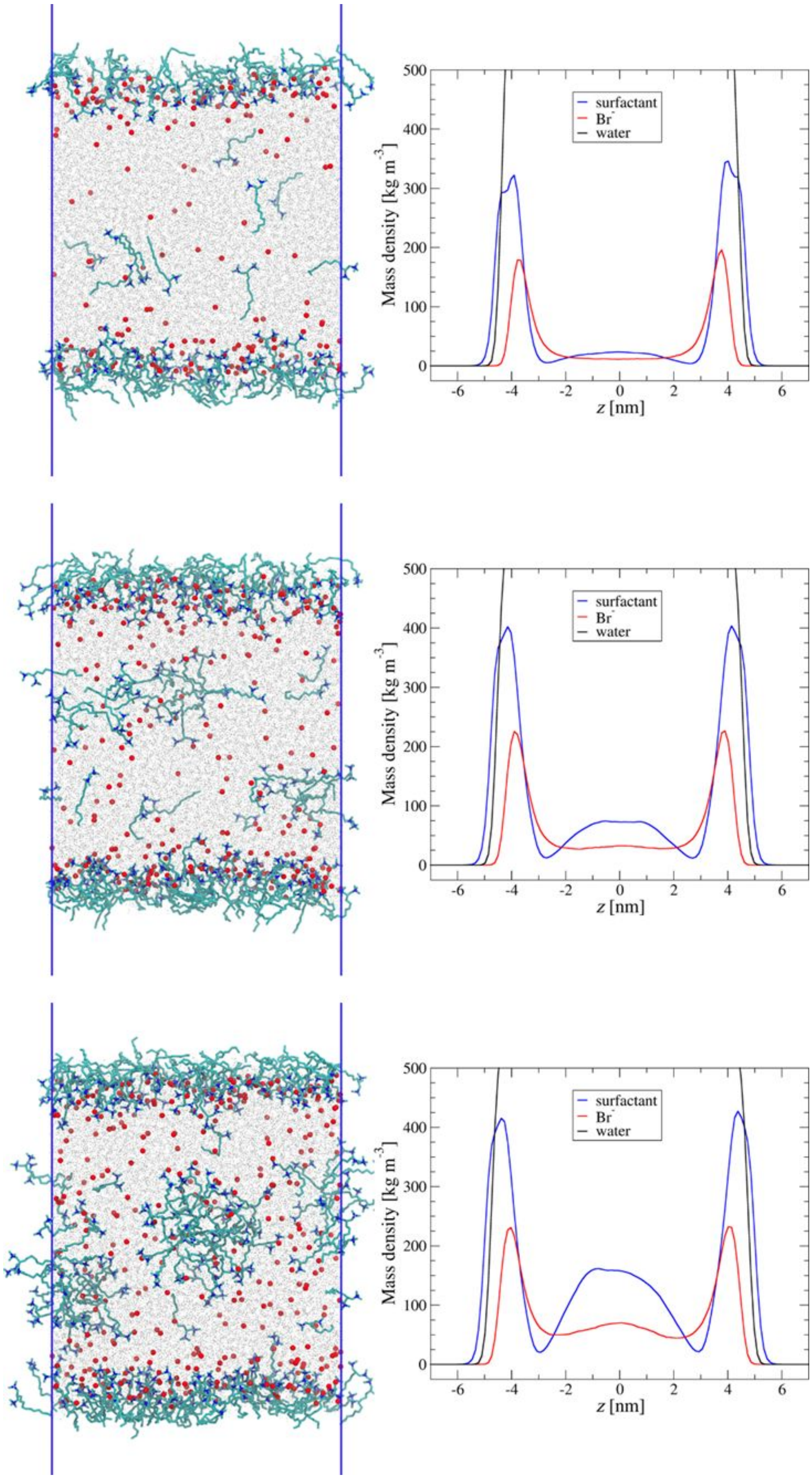

Fig. S5b. Snapshots showing surfactant configurations on gas/water interface after 350 ns of MD simulations. On the right, the mass density distribution across the *z*-direction. From top to bottom: surface concentrations 1.17×10^{-6} mol/m^3 , 1.49×10^{-6} mol/m^3, and 1.6×10^{-6} mol/m^3.

8. CTAB surface tension isotherms in the presence of salts

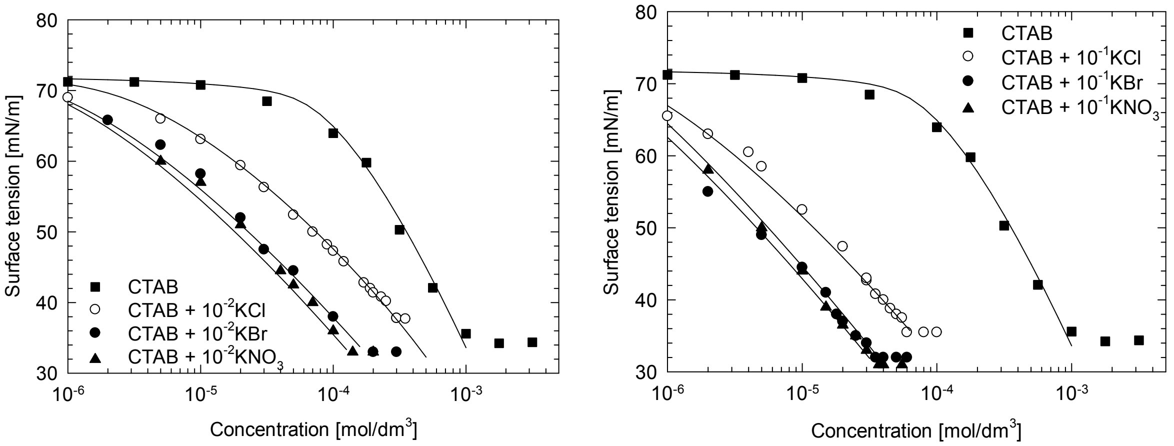

Fig. S6. Surface tension isotherms of CTAB in the presence of 10^{-2} mol/dm^3 and 10^{-2} mol/dm^3 of KBr, KCl, KNO_3. Points - experimental results (in the presence of salts [4]), lines – calculated with the modified STDE model of ionic surfactant adsorption using parameters described in the text.

References

1. Chanachichalermwong, W.; Charoensaeng, A.; Suriyapraphadilok, U. J. Krafft Point Prediction of Anionic Surfactants Using Group Contribution Method: First-order and Higher-order Group. *J. Surfact. Deterg.* **2019**, *22*, 907.
2. Gaussian 16, Revision C.01, M. J. Frisch et al., Gaussian, Inc., Wallingford CT, 2016.
3. H. Yoshida et al., A New Approach to Vibrational Analysis of Large Molecules by Density Functional Theory: Wavenumber-Linear Scaling Method, J. Phys. Chem. A 2002, 106, 3580-3586.
4. G. Para, E. Jarek, P. Warszyński, The Hofmeister series effect in adsorption of cationic surfactants - theoretical description and experimental results, Adv. Colloid Interface Sci**.** **2006**, 122, 39
